# Supplementary material for: Chemical composition and the potential for proteomic transformation in cancer, hypoxia, and hyperosmotic stress
Source: PeerJ. 2017 Jun 6;5:e3421. doi: 10.7717/peerj.3421 (PMC5463988; doi:10.7717/peerj.3421)
Supplement: Figure S1 [file peerj-05-3421-s004.pdf]

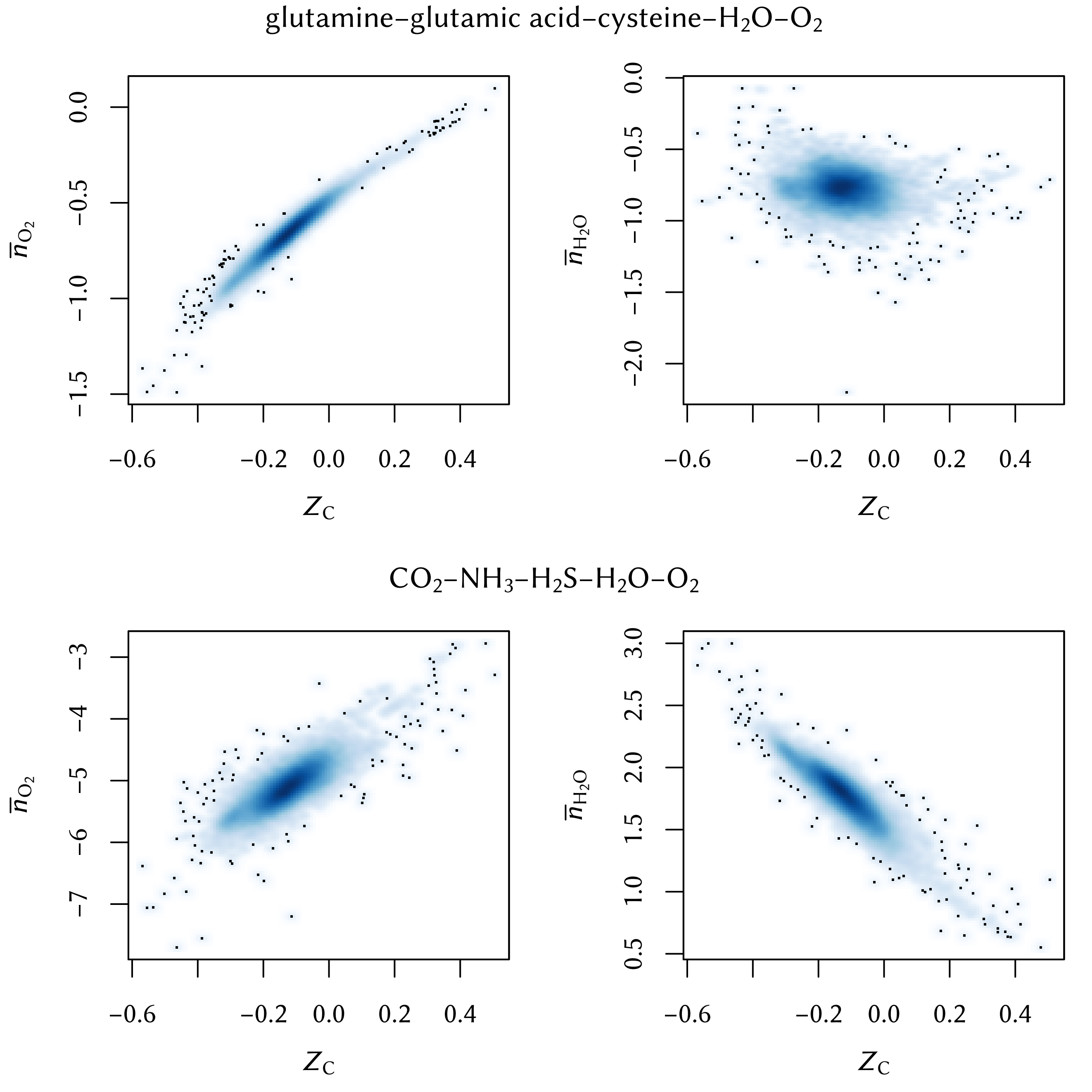
glutamine–glutamic acid–cysteine–H_2O–O_2
$\bar{n}_{O_2}$
$\bar{n}_{H_2O}$
Z_C
CO_2–NH_3–H_2S–H_2O–O_2
$\bar{n}_{O_2}$
$\bar{n}_{H_2O}$
Z_C
