# Supplementary figures and images for: Chemical composition and the potential for proteomic transformation in cancer, hypoxia, and hyperosmotic stress
Source: PeerJ. 2017 Jun 6;5:e3421. doi: 10.7717/peerj.3421 (PMC5463988; doi:10.7717/peerj.3421)

colorectal cancer

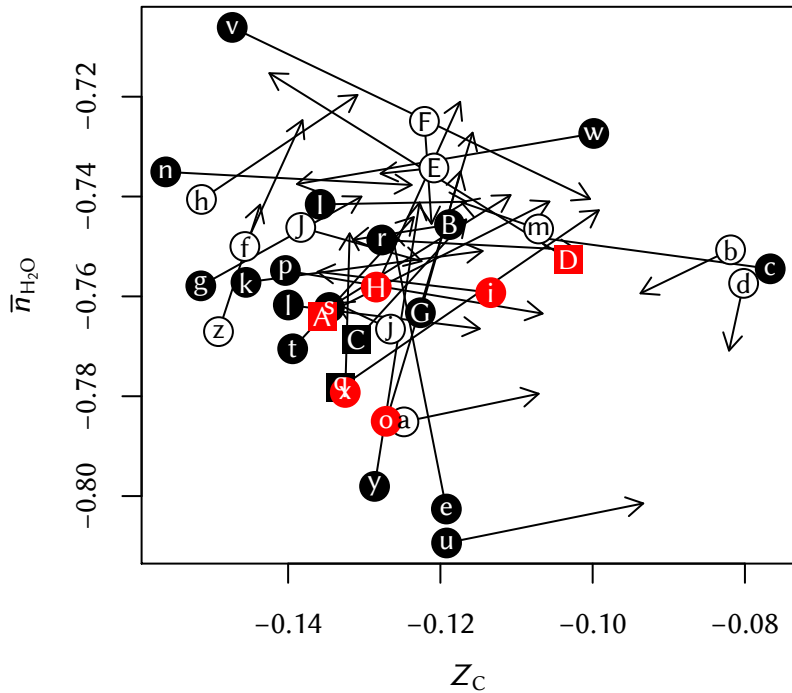

pancreatic cancer

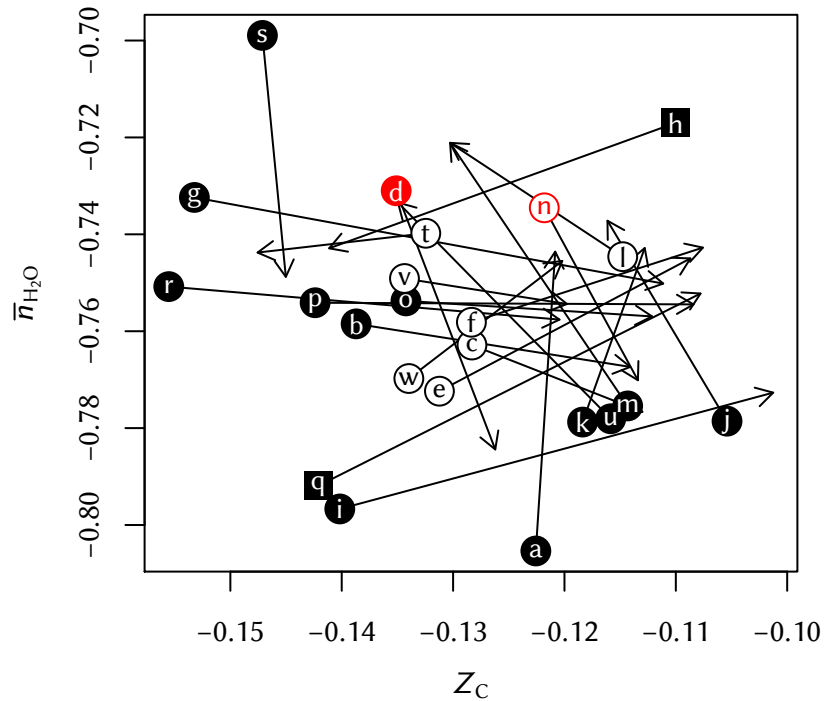

Supplement: Figure S2 [file peerj-05-3421-s005.pdf]
