# Supplementary material for: Chemical composition and the potential for proteomic transformation in cancer, hypoxia, and hyperosmotic stress
Source: PeerJ. 2017 Jun 6;5:e3421. doi: 10.7717/peerj.3421 (PMC5463988; doi:10.7717/peerj.3421)
Supplement: Figure S3 [file peerj-05-3421-s006.pdf]

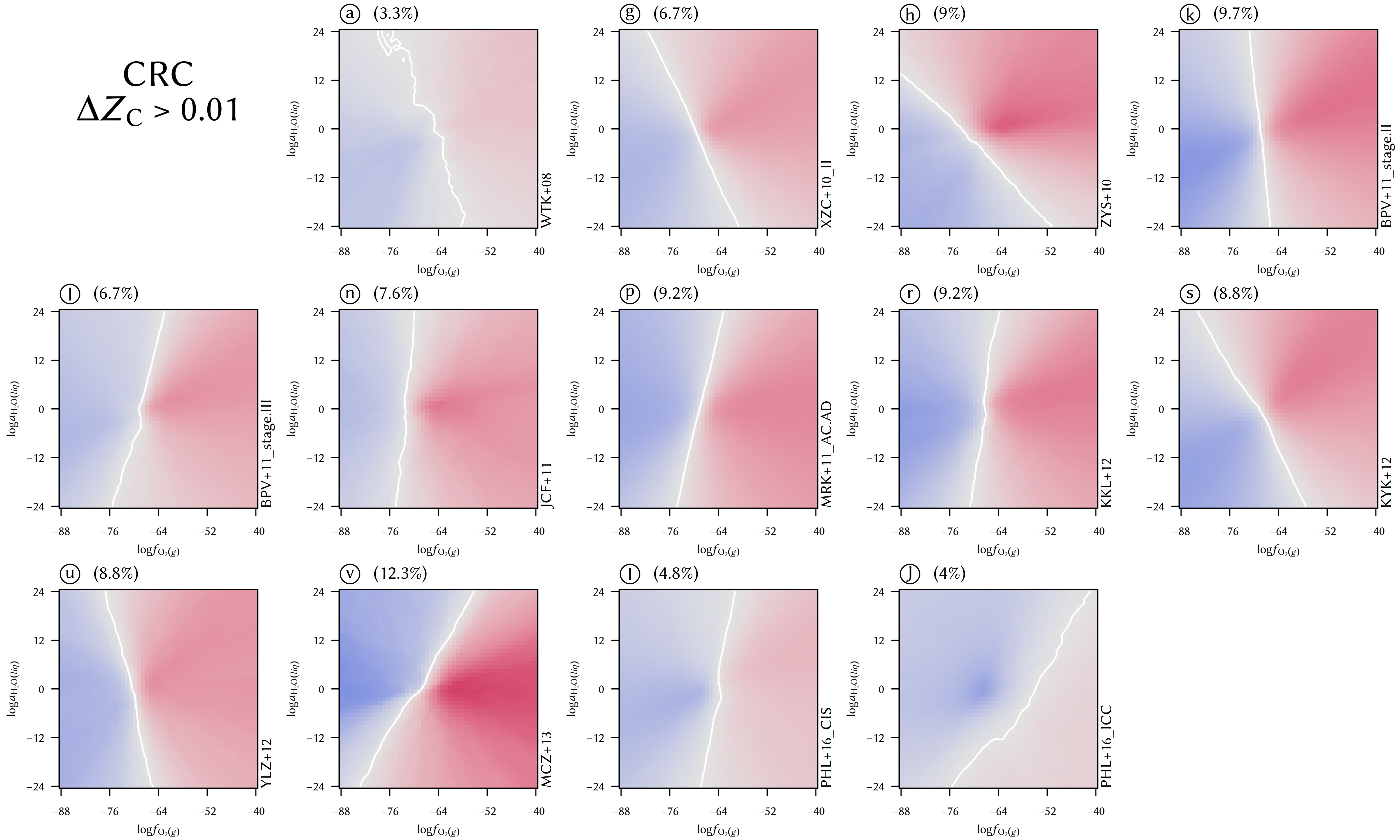

CRC
ΔZC > 0.01
a (3.3%) WTK+08
g (6.7%) XZC+10_II
h (9%) ZYS+10
k (9.7%) BPV+11_stage.II
l (6.7%) BPV+11_stage.III
n (7.6%) JCF+11
p (9.2%) MRK+11_AC.AD
r (9.2%) KKL+12
s (8.8%) KYK+12
u (8.8%) YLZ+12
v (12.3%) MCZ+13
I (4.8%) PHL+16_CIS
J (4%) PHL+16_ICC
logaH2O(liq)
logfO2(g)
24
12
0
−12
−24
−88
−76
−64
−52
−40

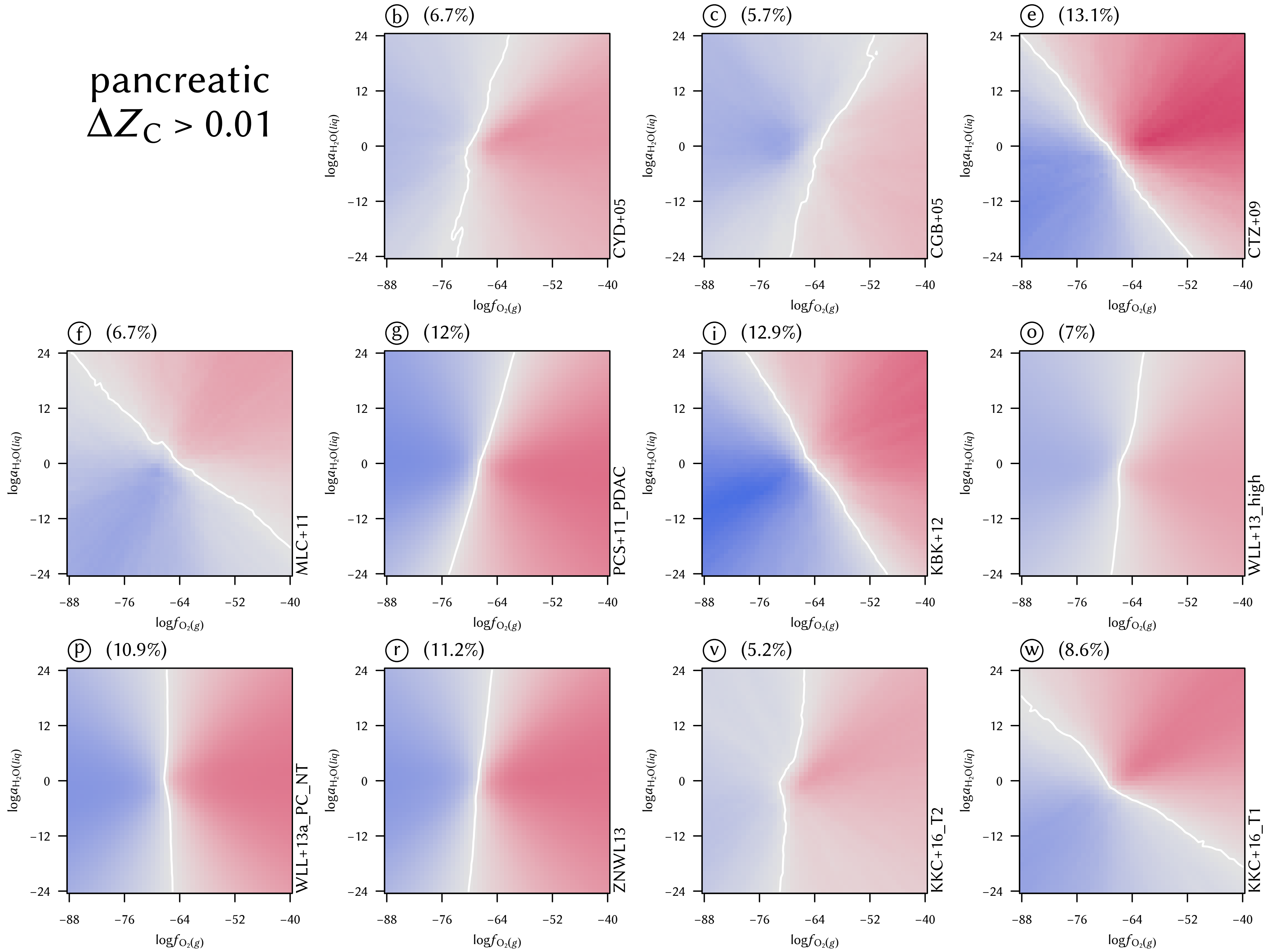
pancreatic
ΔZ_C > 0.01
(b) (6.7%)
CYD+05
(c) (5.7%)
CGB+05
(e) (13.1%)
CTZ+09
(f) (6.7%)
MLC+11
(g) (12%)
PCS+11_PDAC
(i) (12.9%)
KBK+12
(o) (7%)
WLL+13_high
(p) (10.9%)
WLL+13a_PC_NT
(r) (11.2%)
ZNWL13
(v) (5.2%)
KKC+16_T2
(w) (8.6%)
KKC+16_T1
loga_H2O(liq)
logf_O2(g)
24
12
0
−12
−24
−88
−76
−64
−52
−40

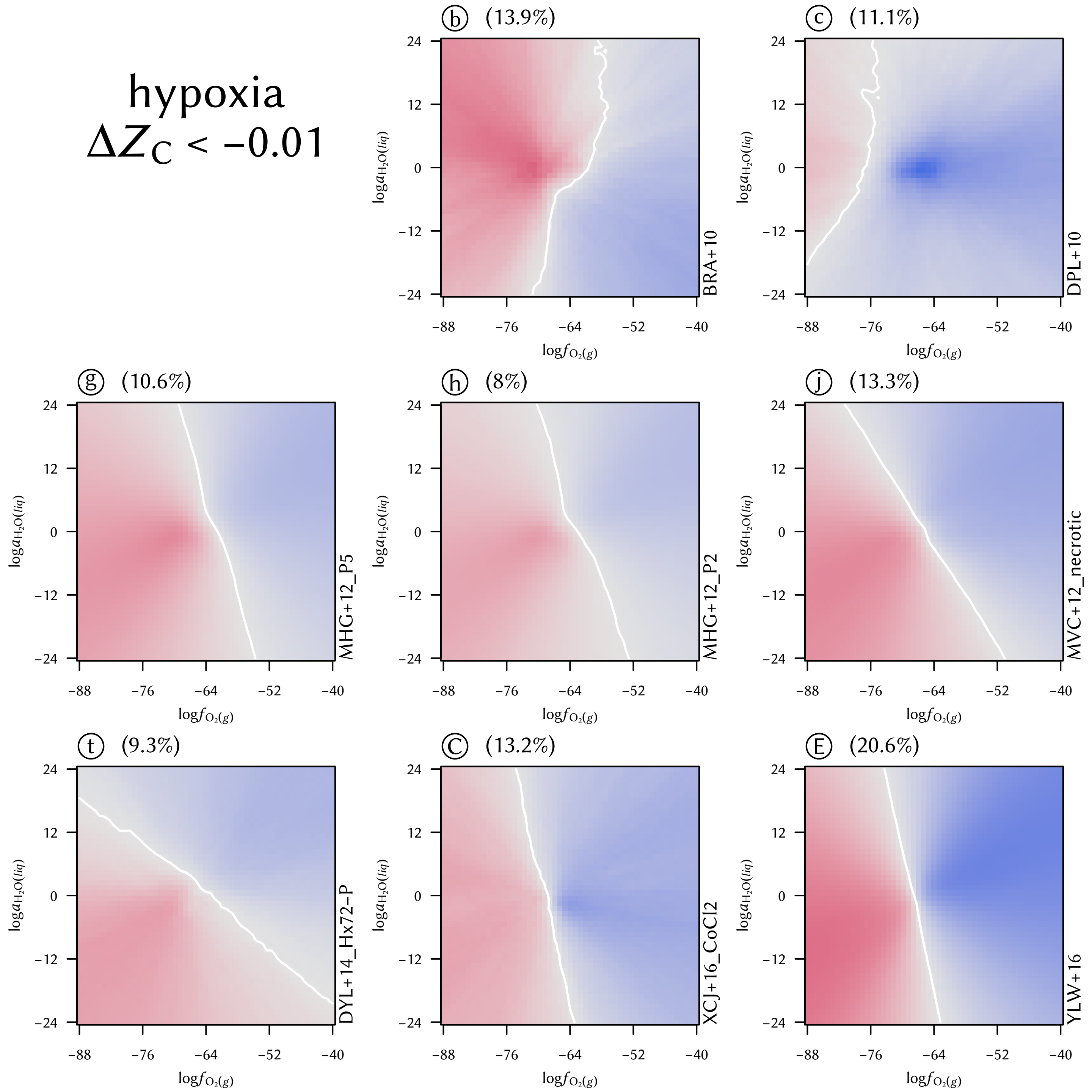

hypoxia
ΔZC < −0.01
b (13.9%)
BRA+10
c (11.1%)
DPL+10
g (10.6%)
MHG+12_P5
h (8%)
MHG+12_P2
j (13.3%)
MVC+12_necrotic
t (9.3%)
DYL+14_Hx72−P
C (13.2%)
XCJ+16_CoCl2
E (20.6%)
YLW+16
loga_H2O(liq)
logf_O2(g)
24
12
0
−12
−24
−88
−76
−64
−52
−40

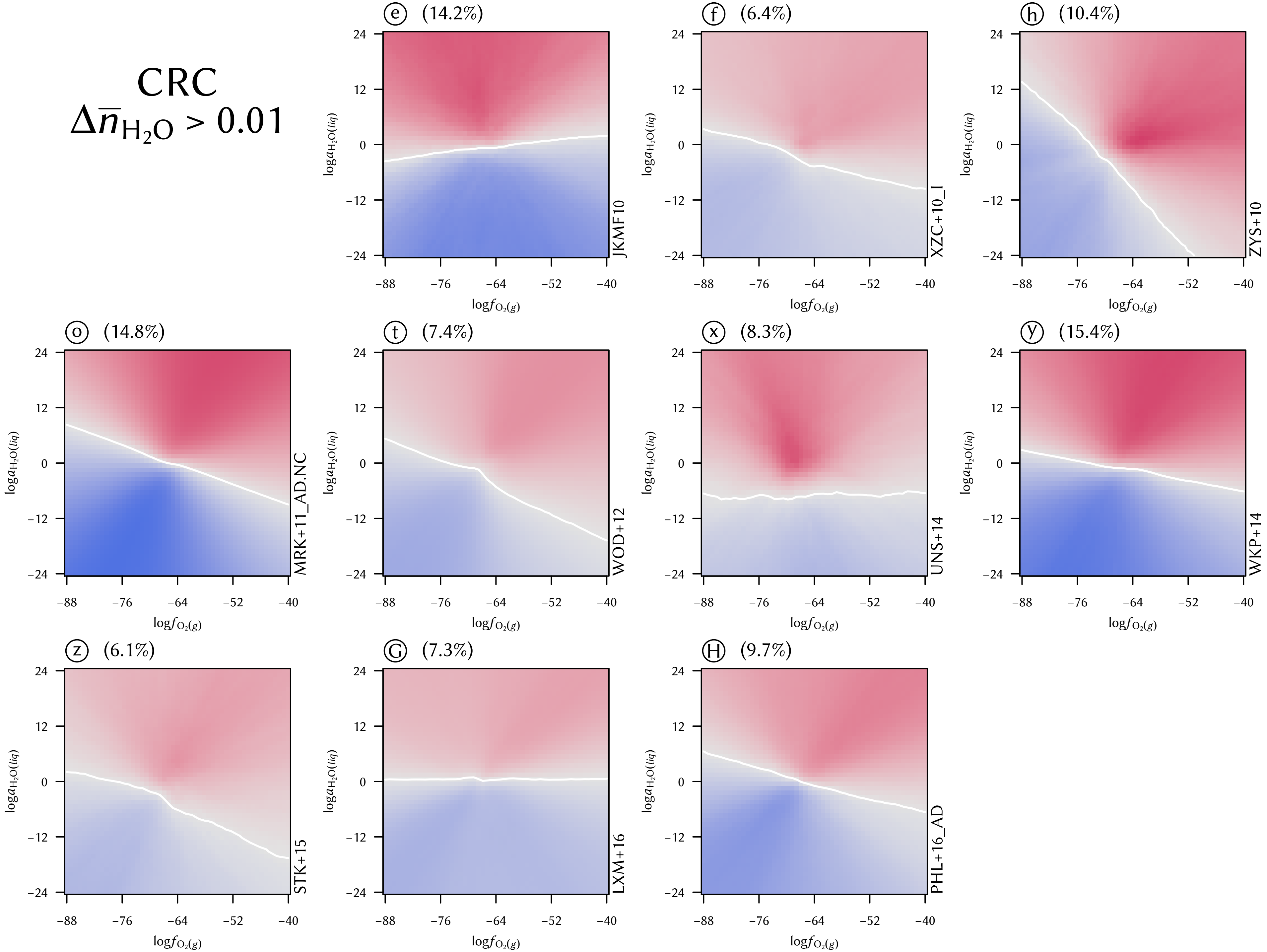

CRC
$\Delta\bar{n}_{H_2O} > 0.01$
e (14.2%)
JKMF10
f (6.4%)
XZC+10_I
h (10.4%)
ZYS+10
o (14.8%)
MRK+11_AD.NC
t (7.4%)
WOD+12
x (8.3%)
UNS+14
y (15.4%)
WKP+14
z (6.1%)
STK+15
G (7.3%)
LXM+16
H (9.7%)
PHL+16_AD
$\log a_{H_2O(liq)}$
$\log f_{O_2(g)}$
24
12
0
−12
−24
−88
−76
−64
−52
−40

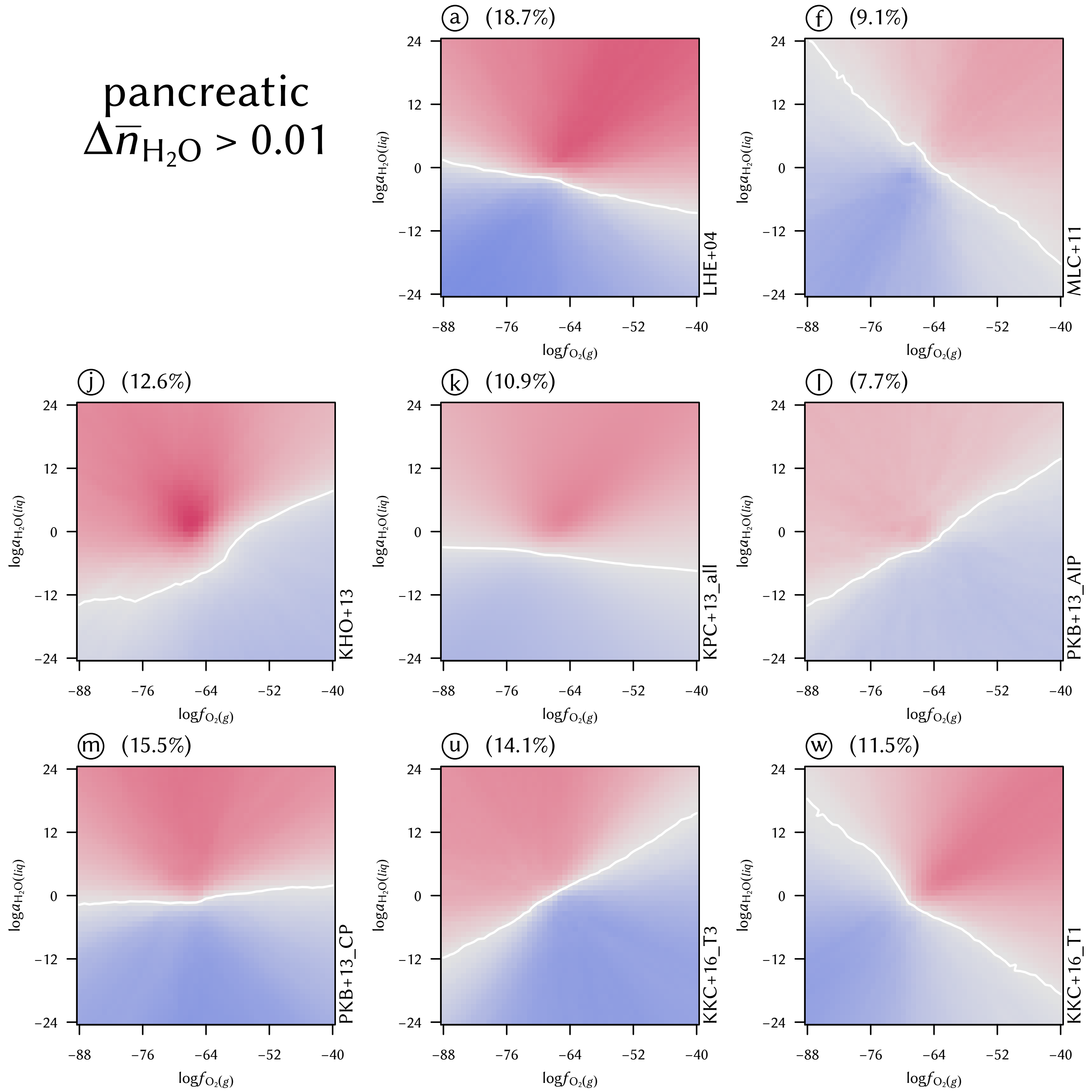
pancreatic
$\Delta\bar{n}_{H_2O} > 0.01$
a (18.7%)
LHE+04
f (9.1%)
MLC+11
j (12.6%)
KHO+13
k (10.9%)
KPC+13_all
l (7.7%)
PKB+13_AIP
m (15.5%)
PKB+13_CP
u (14.1%)
KKC+16_T3
w (11.5%)
KKC+16_T1
$\log a_{H_2O(liq)}$
$\log f_{O_2(g)}$
24
12
0
−12
−24
−88
−76
−64
−52
−40

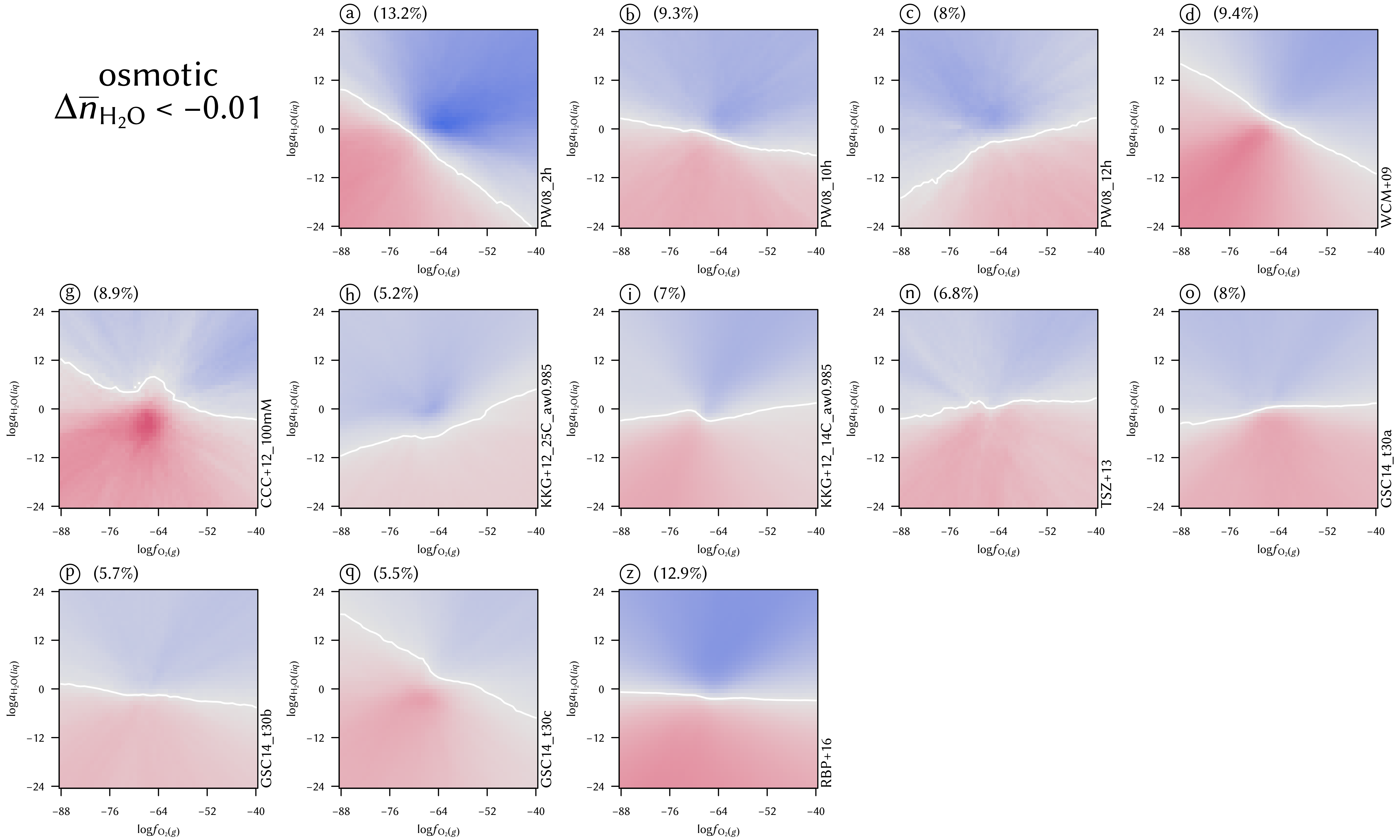

osmotic
$\Delta\bar{n}_{H_2O} < -0.01$
(a) (13.2%) PW08_2h
(b) (9.3%) PW08_10h
(c) (8%) PW08_12h
(d) (9.4%) WCM+09
(g) (8.9%) CCC+12_100mM
(h) (5.2%) KKG+12_25C_aw0.985
(i) (7%) KKG+12_14C_aw0.985
(n) (6.8%) TSZ+13
(o) (8%) GSC14_t30a
(p) (5.7%) GSC14_t30b
(q) (5.5%) GSC14_t30c
(z) (12.9%) RBP+16
$\log a_{H_2O(liq)}$
$\log f_{O_2(g)}$
