# Supplementary material for: Chemical composition and the potential for proteomic transformation in cancer, hypoxia, and hyperosmotic stress
Source: PeerJ. 2017 Jun 6;5:e3421. doi: 10.7717/peerj.3421 (PMC5463988; doi:10.7717/peerj.3421)
Supplement: Dataset S1 [file peerj-05-3421-s001.zip › canprot_0.0.5.tar.gz!/canprot/inst/doc/CRC.html]

Colorectal Cancer


# Colorectal Cancer

This vignette from the R package canprot reproduces calculations of compositional oxidation state and hydration state and thermodynamic potential that are described in a paper published in *PeerJ* (Dick, 2016) and in a preprint posted on bioRxiv (Dick, 2017).

The sections below give the Data Sources for protein expression in colorectal cancer (CRC) contained in this package, a Summary Table comparing the chemical compositions of groups of human proteins that are relatively down- and up-expressed (`n1` and `n2`, respectively) in colorectal cancer, a plot of the Mean Differences of average oxidation state of carbon (`ZC`) and water demand per residue (`nH2O`), Stability Plots showing the weighted rank difference of chemical affinity between down- and up-expressed groups, and References.

## Abbreviations

T (tumor), N (normal), C (carcinoma or adenocarcinoma), A (adenoma), CM (conditioned media), AD (adenomatous colon polyps), CIS (carcinoma in situ), ICC (invasive colonic carcinoma), NC (non-neoplastic colonic mucosa).

## Data Sources

With the exception of `LXM+16`, this text is taken from Dick (2016).

`WTK+08`: Watanabe et al. (2008) used 2-nitrobenzenesulfenyl labeling and MS/MS analysis to identify 128 proteins with differential expression in paired CRC and normal tissue specimens from 12 patients. The list of proteins used in this study was generated by combining the lists of up- and down-regulated proteins from Table 1 and Supplementary Data 1 of Watanabe et al. (2008) with the Swiss-Prot and UniProt accession numbers from their Supplementary Data 2.

`AKP+10`: Albrethsen et al. (2010) used nano-LC-MS/MS to characterize proteins from the nuclear matrix fraction in samples from 2 patients each with adenoma (ADE), chromosomal instability CRC (CIN+) and microsatellite instability CRC (MIN+). Cluster analysis was used to classify proteins with differential expression between ADE and CIN+, MIN+, or in both subtypes of carcinoma (CRC). Here, gene names from Supplementary Tables 5–7 of Albrethsen et al. (2010) were converted to UniProt IDs using the UniProt mapping tool.

`JKMF10`: Jimenez et al. (2010) compiled a list of candidate serum biomarkers from a meta-analysis of the literature. In the meta-analysis, 99 up- or down-expressed proteins were identified in at least 2 studies. The list of UniProt IDs used in this study was taken from Table 4 of Jimenez et al. (2010).

`XZC+10`: Xie et al. (2010) used a gel-enhanced LC-MS method to analyze proteins in pooled tissue samples from 13 stage I and 24 stage II CRC patients and pooled normal colonic tissues from the same patients. Here, IPI accession numbers from Supplemental Table 4 of Xie et al. (2010) were converted to UniProt IDs using the DAVID conversion tool.

`ZYS+10`: Zhang et al. (2010) used acetylation stable isotope labeling and LTQ-FT MS to analyze proteins in pooled microdissected epithelial samples of tumor and normal mucosa from 20 patients, finding 67 and 70 proteins with increased or decreased expression (ratios ≥2 or ≤0.5). Here, IPI accession numbers from Supplemental Table 4 of Zhang et al. (2010) were converted to UniProt IDs using the DAVID conversion tool.

`BPV+11`: Besson et al. (2011) analyzed microdissected cancer and normal tissues from 28 patients (4 adenoma samples and 24 CRC samples at different stages) using iTRAQ labeling and MALDI-TOF/TOF MS to identify 555 proteins with differential expression between adenoma and stage I, II, III, IV CRC. Here, gene names from supplemental Table 9 of Besson et al. (2011) were converted to UniProt IDs using the UniProt mapping tool.

`JCF+11`: Jankova et al. (2011) analyzed paired samples from 16 patients using iTRAQ-MS to identify 118 proteins with >1.3-fold differential expression between CRC tumors and adjacent normal mucosa. The protein list used in this study was taken from Supplementary Table 2 of Jankova et al. (2011).

`MRK+11`: Mikula et al. (2011) used iTRAQ labeling with LC-MS/MS to identify a total of 1061 proteins with differential expression (fold change ≥1.5 and false discovery rate ≤0.01) between pooled samples of 4 normal colon (NC), 12 tubular or tubulo-villous adenoma (AD) and 5 adenocarcinoma (AC) tissues. The list of proteins used in this study was taken from from Table S8 of Mikula et al. (2011).

`KKL+12`: Kim et al. (2012) used difference in-gel electrophoresis (DIGE) and cleavable isotope-coded affinity tag (cICAT) labeling followed by mass spectrometry to identify 175 proteins with more than 2-fold abundance ratios between microdissected and pooled tumor tissues from stage-IV CRC patients with good outcomes (survived more than five years; 3 patients) and poor outcomes (died within 25 months; 3 patients). The protein list used in this study was made by filtering the cICAT data from Supplementary Table 5 of Kim et al. (2012) with an abundance ratio cutoff of >2 or <0.5, giving 147 proteins. IPI accession numbers were converted to UniProt IDs using the DAVID conversion tool.

`KYK+12`: Kang et al. (2012) used mTRAQ and cICAT analysis of pooled microsatellite stable (MSS-type) CRC tissues and pooled matched normal tissues from 3 patients to identify 1009 and 478 proteins in cancer tissue with increased or decreased expression by higher than 2-fold, respectively. Here, the list of proteins from Supplementary Table 4 of Kang et al. (2012) was filtered to include proteins with expression ratio >2 or <0.5 in both mTRAQ and cICAT analyses, leaving 175 up-expressed and 248 down-expressed proteins in CRC. Gene names were converted to UniProt IDs using the UniProt mapping tool.

`WOD+12`: Wiśniewski et al. (2012) used LC-MS/MS to analyze proteins in microdissected samples of formalin-fixed paraffin-embedded (FFPE) tissue from 8 patients; at *P* < 0.01, 762 proteins had differential expression between normal mucosa and primary tumors. The list of proteins used in this study was taken from Supplementary Table 4 of Wiśniewski et al. (2012).

`YLZ+12`: Yao et al. (2012) analyzed the conditioned media of paired stage I or IIA CRC and normal tissues from 9 patients using lectin affinity capture for glycoprotein (secreted protein) enrichment by nano LC-MS/MS to identify 68 up-regulated and 55 down-regulated differentially expressed proteins. IPI accession numbers listed in Supplementary Table 2 of Yao et al. (2012) were converted to UniProt IDs using the DAVID conversion tool.

`MCZ+13`: Mu et al. (2013) used laser capture microdissection (LCM) to separate stromal cells from 8 colon adenocarcinoma and 8 non-neoplastic tissue samples, which were pooled and analyzed by iTRAQ to identify 70 differentially expressed proteins. Here, gi numbers listed in Table 1 of Mu et al. (2013) were converted to UniProt IDs using the UniProt mapping tool; FASTA sequences of 31 proteins not found in UniProt were downloaded from NCBI and amino acid compositions were added to human\_extra.csv.

`KWA+14`: Knol et al. (2014) used differential biochemical extraction to isolate the chromatin-binding fraction in frozen samples of colon adenomas (3 patients) and carcinomas (5 patients), and LC-MS/MS was used for protein identification and label-free quantification. The results were combined with a database search to generate a list of 106 proteins with nuclear annotations and at least a three-fold expression difference. Here, gene names from Table 2 of Knol et al. (2014) were converted to UniProt IDs.

`UNS+14`: Uzozie et al. (2014) analyzed 30 samples of colorectal adenomas and paired normal mucosa using iTRAQ labeling, OFFGEL electrophoresis and LC-MS/MS. 111 proteins with expression fold changes (log_2_) at least +/- 0.5 and statistical significance threshold *q* < 0.02 that were also quantified in cell-line experiments were classified as “epithelial cell signature proteins”. UniProt IDs were taken from Table III of Uzozie et al. (2014).

`WKP+14`: de Wit et al. (2014) analyzed the secretome of paired CRC and normal tissue from 4 patients, adopting a five-fold enrichment cutoff for identification of candidate biomarkers. Here, the list of proteins from Supplementary Table 1 of Wit et al. (2014) was filtered to include those with at least five-fold greater or lower abundance in CRC samples and *p* < 0.05. Two proteins listed as “Unmapped by Ingenuity” were removed, and gene names were converted to UniProt IDs using the UniProt mapping tool.

`STK+15`: Sethi et al. (2015) analyzed the membrane-enriched proteome from tumor and adjacent normal tissues from 8 patients using label-free nano-LC-MS/MS to identify 184 proteins with a fold change > 1.5 and *p*-value < 0.05. Here, protein identifiers from Supporting Table 2 of Sethi et al. (2015) were used to find the corresponding UniProt IDs.

`WDO+15`: Wiśniewski et al. (2015) analyzed 8 matched formalin-fixed and paraffin-embedded (FFPE) samples of normal tissue (N) and adenocarcinoma (C) and 16 nonmatched adenoma samples (A) using LC-MS to identify 2300 (N/A), 1780 (A/C) and 2161 (N/C) up- or down-regulated proteins at *p* < 0.05. The list of proteins used in this study includes only those marked as having a significant change in SI Table 3 of Wiśniewski et al. (2015).

`LPL+16`: Li et al. (2016) used iTRAQ and 2D LC-MS/MS to analyze pooled samples of stroma purified by laser capture microdissection (LCM) from 5 cases of non-neoplastic colonic mucosa (NC), 8 of adenomatous colon polyps (AD), 5 of colon carcinoma in situ (CIS) and 9 of invasive colonic carcinoma (ICC). A total of 222 differentially expressed proteins between NC and other stages were identified. Here, gene symbols from Supplementary Table S3 of Li et al. (2016) were converted to UniProt IDs using the UniProt mapping tool.

`PHL+16`: Peng et al. (2016) used iTRAQ 2D LC-MS/MS to analyze pooled samples from 5 cases of normal colonic mucosa (NC), 8 of adenoma (AD), 5 of carcinoma in situ (CIS) and 9 of invasive colorectal cancer (ICC). A total of 326 proteins with differential expression between two successive stages (and, for CIS and ICC, also differentially expressed with respect to NC) were detected. The list of proteins used in this study was generated by converting the gene names in Supplementary Table 4 of Peng et al. (2016) to UniProt IDs using the UniProt mapping tool.

`LXM+16`: Data were extracted from SI Table S3 of Liu et al. (2016), including proteins with *p*-value <0.05.

## Summary Table

```
library(canprot)
data(canprot)
```

Identify the datasets for protein expression.

```
datasets <- pdat_CRC()
```

Get the amino acid compositions of the up- and down-expressed proteins (`get_pdat`) and make comparisons of indicators of oxidation and hydration state (`ZC_nH2O`).

```
comptab <- lapply_canprot(datasets, function(dataset) {
  pdat <- get_pdat(dataset)
  ZC_nH2O(pdat, plot.it=FALSE)
})
```

Generate the HTML table with `xsummary`, which adds bold and underline formatting to the output of `xtable`. The columns show the difference in means (`DM`), common language effect size (`ES`), and *p*-value (`p`) for comparisons between groups of the average oxidation state of carbon (`ZC`) and water demand per residue (`nH2O`).

```
library(xtable)
xsummary(comptab)
```


|  | | | | *Z*_C_** | | | *n*_H2O_** | | |
| --- | --- | --- | --- | --- | --- | --- | --- | --- | --- |
| set | reference (description) | *n*_1_ | *n*_2_ | MD | ES | *p*-value | MD | ES | *p*-value |
| a | `WTK+08` (T / N) | 57 | 70 | 0.018 | 55 | 3e-01 | 0.006 | 52 | 7e-01 |
| b | `AKP+10` (CRC nuclear matrix C / A) | 101 | 28 | -0.012 | 47 | 7e-01 | -0.009 | 48 | 8e-01 |
| c | `AKP+10` (CIN nuclear matrix C / A) | 87 | 81 | **-0.031** | **40** | **3e-02** | 0.006 | 48 | 7e-01 |
| d | `AKP+10` (MIN nuclear matrix C / A) | 157 | 76 | -0.002 | 52 | 7e-01 | -0.013 | 45 | 3e-01 |
| e | `JKMF10` (serum biomarkers up / down) | 43 | 56 | -0.007 | 46 | 5e-01 | **0.056** | **67** | **4e-03** |
| f | `XZC+10` (stage I / normal) | 48 | 166 | 0.008 | 52 | 7e-01 | 0.025 | 56 | 2e-01 |
| g | `XZC+10` (stage II / normal) | 77 | 321 | **0.021** | **60** | **7e-03** | 0.018 | 54 | 3e-01 |
| h | `ZYS+10` (microdissected T / N) | 61 | 57 | 0.020 | 59 | 1e-01 | 0.021 | 58 | 2e-01 |
| i | `BPV+11` (adenoma / normal) | 71 | 92 | **-0.023** | **40** | **4e-02** | 0.004 | 49 | 8e-01 |
| j | `BPV+11` (stage I / normal) | 109 | 72 | -0.007 | 47 | 5e-01 | 0.005 | 50 | 9e-01 |
| k | `BPV+11` (stage II / normal) | 164 | 140 | **0.031** | **62** | **3e-04** | 0.006 | 51 | 7e-01 |
| l | `BPV+11` (stage III / normal) | 63 | 131 | **0.025** | **62** | **9e-03** | -0.005 | 47 | 5e-01 |
| m | `BPV+11` (stage IV / normal) | 42 | 26 | -0.010 | 44 | 4e-01 | 0.005 | 52 | 8e-01 |
| n | `JCF+11` (T / N) | 72 | 45 | **0.032** | **63** | **2e-02** | -0.003 | 49 | 8e-01 |
| o | `MRK+11` (adenoma / normal) | 335 | 288 | 0.011 | 54 | 1e-01 | **0.058** | **68** | **2e-15** |
| p | `MRK+11` (adenocarcinoma / adenoma) | 373 | 257 | **0.034** | **65** | **1e-10** | -0.009 | 47 | 1e-01 |
| q | `MRK+11` (adenocarcinoma / normal) | 351 | 232 | **0.034** | **63** | **4e-08** | **0.035** | **61** | **8e-06** |
| r | `KKL+12` (poor / good prognosis) | 75 | 61 | **0.026** | **64** | **5e-03** | -0.002 | 48 | 7e-01 |
| s | `KYK+12` (MSS-type T / N) | 73 | 175 | **0.024** | **61** | **9e-03** | 0.023 | 56 | 1e-01 |
| t | `WOD+12` (T / N) | 79 | 677 | 0.016 | 54 | 2e-01 | 0.026 | 58 | **2e-02** |
| u | `YLZ+12` (CM T / N) | 55 | 68 | **0.026** | **62** | **2e-02** | 0.008 | 53 | 6e-01 |
| v | `MCZ+13` (stromal T / N) | 33 | 37 | **0.047** | **74** | **5e-04** | -0.034 | 42 | 2e-01 |
| w | `KWA+14` (chromatin-binding C / A) | 51 | 55 | **-0.039** | **29** | **2e-04** | -0.010 | 48 | 7e-01 |
| x | `UNS+14` (epithelial adenoma / normal) | 58 | 65 | 0.001 | 49 | 8e-01 | **0.032** | **61** | **4e-02** |
| y | `WKP+14` (tissue secretome T / N) | 44 | 210 | 0.006 | 53 | 6e-01 | **0.057** | **68** | **1e-04** |
| z | `STK+15` (membrane enriched T / N) | 113 | 66 | 0.005 | 52 | 6e-01 | 0.025 | 55 | 2e-01 |
| A | `WDO+15` (adenoma / normal) | 1061 | 1254 | **0.030** | **64** | **6e-33** | 0.023 | 58 | **7e-11** |
| B | `WDO+15` (carcinoma / adenoma) | 772 | 1007 | -0.013 | 42 | **2e-08** | -0.003 | 50 | 7e-01 |
| C | `WDO+15` (carcinoma / normal) | 879 | 1281 | 0.014 | 57 | **9e-08** | 0.024 | 58 | **1e-10** |
| D | `LPL+16` (stromal AD / NC) | 123 | 75 | **-0.039** | **32** | **2e-05** | **0.037** | **60** | **2e-02** |
| E | `LPL+16` (stromal CIS / NC) | 125 | 60 | -0.007 | 46 | 4e-01 | -0.001 | 52 | 7e-01 |
| F | `LPL+16` (stromal ICC / NC) | 99 | 75 | 0.001 | 47 | 6e-01 | -0.021 | 48 | 7e-01 |
| G | `LXM+16` (biopsy T / N) | 191 | 178 | 0.005 | 50 | 9e-01 | 0.028 | 57 | **2e-02** |
| H | `PHL+16` (AD / NC) | 113 | 86 | 0.011 | 54 | 4e-01 | **0.037** | **60** | **2e-02** |
| I | `PHL+16` (CIS / NC) | 169 | 138 | 0.019 | 59 | **5e-03** | 0.001 | 49 | 7e-01 |
| J | `PHL+16` (ICC / NC) | 129 | 100 | 0.016 | 57 | 5e-02 | -0.007 | 46 | 3e-01 |

## Mean Differences

Using data from the table above, this plot shows that the groups of proteins that are relatively up-expressed in colorectal cancer or more advanced cancer stages predominantly have higher `ZC` and/or `nH2O`. The datasets comparing adenoma to normal proteomes are highlighted in red.

```
col <- rep("black", length(datasets))
# highlight adenoma / normal datasets
col[grepl("=AD", datasets)] <- "red"
diffplot(comptab, col=col)
```

## Stability Plots

These plots show the weighted rank-sum differences of chemical affinities of formation from basis species of proteins in different groups. A higher ranking of relatively down- or up-expressed proteins in colorectal cancer is represented by blue or red color, respectively. The last plot shows effective values of Eh (redox potential) as a function of the same variables (oxygen fugacity and water activity).

Identify the datasets for protein expression.

```
datasets <- c("JKMF10", "UNS+14", "WKP+14", "MRK+11_AD.NC", "MRK+11_AC.AD", "MRK+11_AC.NC", "JCF+11", "KWA+14")
```

Set up the diagram and make the plots.

```
par(mfrow=c(3, 3))
par(xaxs="i", yaxs="i", las=1, mar=c(4, 4, 2, 2), mgp=c(2.6, 1, 0), cex=1)
for(i in seq_along(datasets)) {
  pdat <- get_pdat(datasets[i], basis="QEC4")
  rankplot(pdat, res=50)
  CHNOSZ::label.figure(LETTERS[i], paren=FALSE, font=2, yfrac=0.94)
}
Ehplot()
CHNOSZ::label.figure("I", paren=FALSE, font=2, yfrac=0.94)
```

This is similar to Figure 6 of Dick (2016). To reduce the time needed to create the vignette, the plots here are made at lower resolution than those in the paper.

## References

Albrethsen J., Knol JC., Piersma SR., Pham TV., Wit M de., Mongera S., Carvalho B., Verheul HMW., Fijneman RJA., Meijer GA., Jimenez CR. 2010. Subnuclear proteomics in colorectal cancer: Identification of proteins enriched in the nuclear matrix fraction and regulation in adenoma to carcinoma progression. *Molecular & Cellular Proteomics* 9:988–1005. DOI: 10.1074/mcp.M900546-MCP200.

Besson D., Pavageau A-H., Valo I., Bourreau A., Bélanger A., Eymerit-Morin C., Moulière A., Chassevent A., Boisdron-Celle M., Morel A., Solassol J., Campone M., Gamelin E., Barré B., Coqueret O., Guette C. 2011. A quantitative proteomic approach of the different stages of colorectal cancer establishes OLFM4 as a new nonmetastatic tumor marker. *Molecular & Cellular Proteomics* 10:M111.009712. DOI: 10.1074/mcp.M111.009712.

Jankova L., Chan C., Fung CLS., Song X., Kwun SY., Cowley MJ., Kaplan W., Dent OF., Bokey EL., Chapuis PH., Baker MS., Robertson GR., Clarke SJ., Molloy MP. 2011. Proteomic comparison of colorectal tumours and non-neoplastic mucosa from paired patient samples using iTRAQ mass spectrometry. *Molecular Biosystems* 7:2997–3005. DOI: 10.1039/C1MB05236E.

Jimenez CR., Knol JC., Meijer GA., Fijneman RJA. 2010. Proteomics of colorectal cancer: Overview of discovery studies and identification of commonly identified cancer-associated proteins and candidate CRC serum markers. *Journal of Proteomics* 73:1873–1895. DOI: 10.1016/j.jprot.2010.06.004.

Kang U-B., Yeom J., Kim H-J., Kim H., Lee C. 2012. Expression profiling of more than 3500 proteins of MSS-type colorectal cancer by stable isotope labeling and mass spectrometry. *Journal of Proteomics* 75:3050–3062. DOI: 10.1016/j.jprot.2011.11.021.

Kim H-J., Kang U-B., Lee H., Jung J-H., Lee S-T., Yu M-H., Kim H., Lee C. 2012. Profiling of differentially expressed proteins in stage IV colorectal cancers with good and poor outcomes. *Journal of Proteomics* 75:2983–2997. DOI: 10.1016/j.jprot.2011.12.002.

Knol JC., Wit M de., Albrethsen J., Piersma SR., Pham TV., Mongera S., Carvalho B., Fijneman RJA., Meijer GA., Jiménez CR. 2014. Proteomics of differential extraction fractions enriched for chromatin-binding proteins from colon adenoma and carcinoma tissues. *Biochimica et Biophysica Acta (BBA) - Proteins and Proteomics* 1844:1034–1043. DOI: 10.1016/j.bbapap.2013.12.006.

Li M., Peng F., Li G., Fu Y., Huang Y., Chen Z., Chen Y. 2016. Proteomic analysis of stromal proteins in different stages of colorectal cancer establishes Tenascin-C as a stromal biomarker for colorectal cancer metastasis. *Oncotarget* 7:37226–37237. DOI: 10.18632/oncotarget.9362.

Liu X., Xu Y., Meng Q., Zheng Q., Wu J., Wang C., Jia W., Figeys D., Chang Y., Zhou H. 2016. Proteomic analysis of minute amount of colonic biopsies by enteroscopy sampling. *Biochemical and Biophysical Research Communications* 476:286–292. DOI: 10.1016/j.bbrc.2016.05.114.

Mikula M., Rubel T., Karczmarski J., Goryca K., Dadlez M., Ostrowski J. 2011. Integrating proteomic and transcriptomic high-throughput surveys for search of new biomarkers of colon tumors. *Functional and Integrative Genomics* 11:215–224. DOI: 10.1007/s10142-010-0200-5.

Mu Y., Chen Y., Zhang G., Zhan X., Li Y., Liu T., Li G., Li M., Xiao Z., Gong X., Chen Z. 2013. Identification of stromal differentially expressed proteins in the colon carcinoma by quantitative proteomics. *Electrophoresis* 34:1679–1692. DOI: 10.1002/elps.201200596.

Peng F., Huang Y., Li M-Y., Li G-Q., Huang H-C., Guan R., Chen Z-C., Liang S-P., Chen Y-H. 2016. Dissecting characteristics and dynamics of differentially expressed proteins during multistage carcinogenesis of human colorectal cancer. *World Journal of Gastroenterology* 22:4515–4528. DOI: 10.3748/wjg.v22.i18.4515.

Sethi MK., Thaysen-Andersen M., Kim H., Park CK., Baker MS., Packer NH., Paik Y-K., Hancock WS., Fanayan S. 2015. Quantitative proteomic analysis of paired colorectal cancer and non-tumorigenic tissues reveals signature proteins and perturbed pathways involved in CRC progression and metastasis. *Journal of Proteomics* 126:54–67. DOI: 10.1016/j.jprot.2015.05.037.

Uzozie A., Nanni P., Staiano T., Grossmann J., Barkow-Oesterreicher S., Shay JW., Tiwari A., Buffoli F., Laczko E., Marra G. 2014. Sorbitol dehydrogenase overexpression and other aspects of dysregulated protein expression in human precancerous colorectal neoplasms: A quantitative proteomics study. *Molecular & Cellular Proteomics* 13:1198–1218. DOI: 10.1074/mcp.M113.035105.

Watanabe M., Takemasa I., Kawaguchi N., Miyake M., Nishimura N., Matsubara T., Matsuo E-i., Sekimoto M., Nagai K., Matsuura N., Monden M., Nishimura O. 2008. An application of the 2-nitrobenzenesulfenyl method to proteomic profiling of human colorectal carcinoma: A novel approach for biomarker discovery. *Proteomics: Clinical Applications* 2:925–935. DOI: 10.1002/prca.200780111.

Wiśniewski JR., Ostasiewicz P., Duś K., Zielińska DF., Gnad F., Mann M. 2012. Extensive quantitative remodeling of the proteome between normal colon tissue and adenocarcinoma. *Molecular Systems Biology* 8:611. DOI: 10.1038/msb.2012.44.

Wiśniewski JR., Duś-Szachniewicz K., Ostasiewicz P., Ziółkowski P., Rakus D., Mann M. 2015. Absolute proteome analysis of colorectal mucosa, adenoma, and cancer reveals drastic changes in fatty acid metabolism and plasma membrane transporters. *Journal of Proteome Research* 14:4005–4018. DOI: 10.1021/acs.jproteome.5b00523.

Wit M de., Kant H., Piersma SR., Pham TV., Mongera S., Berkel MPA van., Boven E., Pontén F., Meijer GA., Jimenez CR., Fijneman RJA. 2014. Colorectal cancer candidate biomarkers identified by tissue secretome proteome profiling. *Journal of Proteomics* 99:26–39. DOI: 10.1016/j.jprot.2014.01.001.

Xie L-Q., Zhao C., Cai S-J., Xu Y., Huang L-Y., Bian J-S., Shen C-P., Lu H-J., Yang P-Y. 2010. Novel proteomic strategy reveal combined \(\alpha\)1 antitrypsin and cathepsin D as biomarkers for colorectal cancer early screening. *Journal of Proteome Research* 9:4701–4709. DOI: 10.1021/pr100406z.

Yao L., Lao W., Zhang Y., Tang X., Hu X., He C., Hu X., Xu LX. 2012. Identification of EFEMP2 as a serum biomarker for the early detection of colorectal cancer with lectin affinity capture assisted secretome analysis of cultured fresh tissues. *Journal of Proteome Research* 11:3281–3294. DOI: 10.1021/pr300020p.

Zhang Y., Ye Y., Shen D., Jiang K., Zhang H., Sun W., Zhang J., Xu F., Cui Z., Wang S. 2010. Identification of transgelin-2 as a biomarker of colorectal cancer by laser capture microdissection and quantitative proteome analysis. *Cancer Science* 101:523–529. DOI: 10.1111/j.1349-7006.2009.01424.x.
