# Supplementary material for: Chemical composition and the potential for proteomic transformation in cancer, hypoxia, and hyperosmotic stress
Source: PeerJ. 2017 Jun 6;5:e3421. doi: 10.7717/peerj.3421 (PMC5463988; doi:10.7717/peerj.3421)
Supplement: Dataset S1 [file peerj-05-3421-s001.zip › canprot_0.0.5.tar.gz!/canprot/inst/doc/hypoxia.html]

Hypoxia and 3D Culture


# Hypoxia and 3D Culture

This vignette from the R package canprot reproduces calculations of compositional oxidation state and hydration state that are described in a preprint posted on bioRxiv (Dick, 2017).

## Abbreviations

U937 (acute promonocytic leukemic cells), B104 (rat neuroblastoma cells), DU145 (prostate carcinoma cells), SK-N-BE(2)c; IMR-32; SH-SY5Y (neuroblastoma cells), H9C2 (rat heart myoblast), MCF-7 (breast cancer cells), THP-1 (macrophages), A431 (epithelial carcinoma cells), Hx48 (hypoxia 48 h), Hx72 (hypoxia 72 h), ReOx (hypoxia 48 h followed by reoxygenation for 24 h), -S (supernatant fraction), -P (pellet fraction), SPH (spheroids), HepG2/C3A (hepatocellular carcinoma cells), U87MG (glioblastoma), 786-O (renal clear cell carcinoma cells), HCT116; HT29 (colon cancer cells), SC (stem cells), SAL (salidroside).

## Data Sources

`HXS+06` 2% O_2_ vs normoxic conditions. Source: Table 1 of Han et al. (2006).

`BRA+10` 1% vs 6% O_2_. Source: Tables 2 and 3 of Blankley et al. (2010).

`DPL+10` The comparisons here use expression ratios HYP/LSC (oxygen deprivation / low serum control) >1.2 or <0.83, calculated from the reported ratios. Source: extracted from Suppl. Table 2 of Datta et al. (2010), including proteins with *p*-value <0.05 and EF <1.4.

`BMJ+11` Translationally regulated genes. Source: Suppl. Tables 1–4 of Beucken et al. (2011).

`CBW+11` 1% O_2_ for 72 h vs standard conditions. Source: Suppl. Table 1(a) of Cifani et al. (2011).

`LAR+12` Hypoxic vs control conditions for 16 h. Source: Suppl. Table S5 of Li et al. (2012).

`MHG+12` Tumourspheres (50 to 200 μm diameter) at passage 5 (P5) or 2 (P2) compared to adherent cells. Source: Sheets 2 and 3 in Table S1 of Morrison et al. (2012).

`MVC+12` Perinecrotic and necrotic regions compared to surface of multicell spheroids (~600 μm diameter). The comparisons here use expression ratios <0.77 or >1.3. Source: Suppl. Table 1C of McMahon et al. (2012).

`FWH+13` Incubation for several days under hypoxia (1% O_2_). Source: Suppl. Table 2A of Fuhrmann et al. (2013) (control virus cells).

`RHD+13` Source: extracted from Suppl. Table 1 of Ren et al. (2013), including proteins with iTRAQ ratios <0.83 or >1.2 and *p*-value <0.05.

`VTMF13` 5% O_2_ vs atmospheric levels of O_2_. The comparisons here include proteins with a normalized expression ratio of >1.2 or <0.83. Source: SI table of Villeneuve et al. (2013).

`DYL+14` The comparisons here include proteins with *p* <0.05. Source: Suppl. Table S1 of Dutta et al. (2014).

`RKP+14` Organotypic spheroids (~250 μm diameter) vs lysed CRC tissue. Source: extracted from Table S2 of Rajcevic et al. (2014), filtered as follows: at least two of three experiments have differences in spectral counts, absolute overall fold change is at least 1.5, and *p*-value is less than 0.05.

`WRK+14` SPH vs classical cell culture (2D growth). Standard concentrations of gases used for tissue culture (5% CO_2_, 95% air) were used in both cases. The comparisons here include proteins that have a log_2_ fold change of at least ±1. Source: P1\_Data sheet in the SI of Wrzesinski et al. (2014).

`BSA+15` 1% vs 19% O_2_. Source: Table S1 of Bousquet et al. (2015).

`HWA+16` 1% O_2_ for 24 hr. The comparisons here include proteins with a fold change of <0.5 or >1 and proteins that were detected in only hypoxic or only normoxic conditions. Source: Table S1 of Ho et al. (2016).

`LCS16` Microarray analysis of differential gene expression in the transcriptome (total rRNA) and translatome (polysomal / total RNA ratio) of cells grown in normal and hypoxic (1% O_2_) conditions. Source: data file supplied by Dr. Ming-Chih Lai (Lai, Chang & Sun (2016)).

`RSE+16` ASC from 3 donors cultured for 24 hr. in hypoxic (1% O_2_) vs normoxic (20% O_2_) conditions. Source: Tables 1 and 2 of Riis et al. (2016).

`XCJ+16` Rat cardiomyocytes treated with CoCl_2_ (hypoxia mimetic) vs control or with SAL (anti-hypoxic) vs CoCl_2_. Source: SI Tables 1S and 2S of Xu et al. (2016).

`YLW+16` 800 μm spheroids vs 2D monolayers. Source: Tables S1a–b of Yue et al. (2016).

## Summary Table

This table compares the chemical compositions of groups of proteins that are relatively down- and up-expressed (`n1` and `n2`, respectively) in cells grown in hypoxia or 3D culture compared to control conditions.

```
library(canprot)
data(canprot)
```

```
datasets <- pdat_hypoxia()
```

```
comptab <- lapply_canprot(datasets, function(dataset) {
  pdat <- get_pdat(dataset, "pdat_hypoxia")
  ZC_nH2O(pdat, plot.it=FALSE)
}, varlist="pdat_hypoxia")
```

```
library(xtable)
xsummary(comptab)
```


|  | | | | *Z*_C_** | | | *n*_H2O_** | | |
| --- | --- | --- | --- | --- | --- | --- | --- | --- | --- |
| set | reference (description) | *n*_1_ | *n*_2_ | MD | ES | *p*-value | MD | ES | *p*-value |
| a | `HXS+06` (U937) | 37 | 24 | **-0.027** | **31** | **1e-02** | 0.029 | **62** | 1e-01 |
| b | `BRA+10` (placental secretome) | 41 | 22 | -0.027 | **38** | 1e-01 | 0.011 | 54 | 6e-01 |
| c | `DPL+10` (B104) | 71 | 19 | -0.021 | **37** | 9e-02 | 0.001 | 51 | 9e-01 |
| d | `BMJ+11` (DU145) | 87 | 28 | 0.014 | **61** | 8e-02 | 0.000 | 49 | 9e-01 |
| e | `CBW+11` (SK-N-BE(2)c; IMR-32) | 29 | 21 | 0.004 | 51 | 9e-01 | 0.001 | 50 | 1e+00 |
| f | `LAR+12` (H9C2) | 53 | 65 | -0.007 | 51 | 8e-01 | **-0.066** | **35** | **6e-03** |
| g | `MHG+12` (MCF-7 SPH P5) | 409 | 337 | **-0.027** | **40** | **1e-06** | -0.008 | 47 | 2e-01 |
| h | `MHG+12` (MCF-7 SPH P2) | 248 | 214 | -0.025 | 42 | **2e-03** | -0.003 | 48 | 5e-01 |
| i | `MVC+12` (SPH perinecrotic) | 48 | 52 | -0.006 | 47 | 6e-01 | -0.025 | 44 | 3e-01 |
| j | `MVC+12` (SPH necrotic) | 101 | 186 | **-0.023** | **38** | **9e-04** | -0.029 | 43 | 6e-02 |
| k | `FWH+13` (THP-1) | 56 | 40 | 0.003 | 53 | 6e-01 | 0.011 | 54 | 5e-01 |
| l | `RHD+13` (A431 Hx48) | 178 | 77 | 0.012 | 53 | 4e-01 | -0.017 | 46 | 4e-01 |
| m | `RHD+13` (A431 Hx72) | 69 | 54 | **-0.025** | **38** | **2e-02** | **-0.048** | **40** | **5e-02** |
| n | `RHD+13` (A431 ReOx) | 48 | 36 | 0.001 | 51 | 8e-01 | 0.003 | 56 | 4e-01 |
| o | `VTMF13` (SH-SY5Y) | 141 | 64 | **-0.021** | **39** | **1e-02** | 0.024 | 59 | **3e-02** |
| p | `DYL+14` (A431 Hx48-S) | 65 | 34 | **0.033** | **65** | **2e-02** | **-0.032** | **37** | **3e-02** |
| q | `DYL+14` (A431 Hx72-S) | 137 | 61 | -0.006 | 49 | 8e-01 | -0.012 | 45 | 2e-01 |
| r | `DYL+14` (A431 ReOx-S) | 56 | 49 | **0.037** | **67** | **4e-03** | **-0.050** | **33** | **2e-03** |
| s | `DYL+14` (A431 Hx48-P) | 74 | 44 | -0.002 | 52 | 8e-01 | -0.008 | 46 | 5e-01 |
| t | `DYL+14` (A431 Hx72-P) | 67 | 53 | -0.013 | 46 | 4e-01 | -0.015 | 43 | 2e-01 |
| u | `DYL+14` (A431 ReOx-P) | 41 | 31 | **0.021** | **64** | **4e-02** | -0.035 | **37** | 6e-02 |
| v | `RKP+14` (CRC-derived SPH) | 113 | 154 | 0.012 | 55 | 2e-01 | 0.005 | 50 | 1e+00 |
| w | `WRK+14` (HepG2/C3A SPH) | 127 | 292 | **-0.032** | **39** | **4e-04** | -0.029 | 43 | **2e-02** |
| x | `BSA+15` (HeLa) | 53 | 72 | 0.004 | 53 | 6e-01 | -0.003 | 50 | 1e+00 |
| y | `HWA+16` (U87MG and 786-O) | 137 | 164 | -0.001 | 49 | 8e-01 | -0.026 | 42 | **2e-02** |
| z | `LCS16` (HCT116 transcription) | 129 | 141 | -0.004 | 47 | 4e-01 | 0.024 | 57 | **4e-02** |
| A | `LCS16` (HCT116 translation) | 469 | 1024 | **-0.028** | **39** | **1e-11** | -0.025 | 43 | **2e-05** |
| B | `RSE+16` (adipose-derived SC) | 66 | 50 | **0.031** | **67** | **2e-03** | -0.011 | 45 | 3e-01 |
| C | `XCJ+16` (cardiomyocytes CoCl2) | 65 | 27 | -0.025 | 41 | 2e-01 | -0.012 | 46 | 6e-01 |
| D | `XCJ+16` (cardiomyocytes SAL) | 35 | 69 | 0.014 | 58 | 2e-01 | -0.004 | 46 | 5e-01 |
| E | `YLW+16` (HT29 SPH) | 116 | 225 | **-0.053** | **29** | **2e-10** | -0.018 | 45 | 2e-01 |

## Mean Differences

The reoxygenation or anti-hypoxic, tumor spheroid, and adipose-derived stem cell datasets are highlighted in blue, red, and orange, respectively.

```
col <- rep("black", length(datasets))
col[grepl("ReOx", datasets)] <- "blue"
col[grepl("=SPH", datasets)] <- "red"
col[grepl("=ASC", datasets)] <- "orange"
diffplot(comptab, col=col)
```

## References

Beucken T van den., Magagnin MG., Jutten B., Seigneuric R., Lambin P., Koritzinsky M., Wouters BG. 2011. Translational control is a major contributor to hypoxia induced gene expression. *Radiotherapy and Oncology* 99:379–384. DOI: 10.1016/j.radonc.2011.05.058.

Blankley RT., Robinson NJ., Aplin JD., Crocker IP., Gaskell SJ., Whetton AD., Baker PN., Myers JE. 2010. A gel-free quantitative proteomics analysis of factors released from hypoxic-conditioned placentae. *Reproductive Sciences* 17:247–257. DOI: 10.1177/1933719109351320.

Bousquet PA., Sandvik JA., Arntzen MØ., Jeppesen Edin NF., Christoffersen S., Krengel U., Pettersen EO., Thiede B. 2015. Hypoxia strongly affects mitochondrial ribosomal proteins and translocases, as shown by quantitative proteomics of HeLa cells. *International Journal of Proteomics* 2015:678527. DOI: 10.1155/2015/678527.

Cifani P., Bendz M., Wårell K., Hansson K., Levander F., Sandin M., Krogh M., Ovenberger M., Fredlund E., Vaapil M., Pietras A., Påhlman S., James P. 2011. Hunting for protein markers of hypoxia by combining plasma membrane enrichment with a new approach to membrane protein analysis. *Journal of Proteome Research* 10:1645–1656. DOI: 10.1021/pr100982j.

Datta A., Park JE., Li X., Zhang H., Ho ZS., Heese K., Lim SK., Tam JP., Sze SK. 2010. Phenotyping of an *in vitro* model of ischemic penumbra by iTRAQ-based shotgun quantitative proteomics. *Journal of Proteome Research* 9:472–484. DOI: 10.1021/pr900829h.

Dutta B., Yan R., Lim SK., Tam JP., Sze SK. 2014. Quantitative profiling of chromatome dynamics reveals a novel role for HP1BP3 in hypoxia-induced oncogenesis. *Molecular & Cellular Proteomics* 13:3236–3249. DOI: 10.1074/mcp.M114.038232.

Fuhrmann DC., Wittig I., Heide H., Dehne N., Brüne B. 2013. Chronic hypoxia alters mitochondrial composition in human macrophages. *Biochimica et Biophysica Acta (BBA) - Proteins and Proteomics* 1834:2750–2760. DOI: 10.1016/j.bbapap.2013.09.023.

Han Y-H., Xia L., Song L-P., Zheng Y., Chen W-L., Zhang L., Huang Y., Chen G-Q., Wang L-S. 2006. Comparative proteomic analysis of hypoxia-treated and untreated human leukemic U937 cells. *Proteomics* 6:3262–3274. DOI: 10.1002/pmic.200500754.

Ho JJD., Wang M., Audas TE., Kwon D., Carlsson SK., Timpano S., Evagelou SL., Brothers S., Gonzalgo ML., Krieger JR., Chen S., Uniacke J., Lee S. 2016. Systemic reprogramming of translation efficiencies on oxygen stimulus. *Cell Reports* 14:1293–1300. DOI: 10.1016/j.celrep.2016.01.036.

Lai M-C., Chang C-M., Sun HS. 2016. Hypoxia induces autophagy through translational up-regulation of lysosomal proteins in human colon cancer cells. *PLoS ONE* 11:1–21. DOI: 10.1371/journal.pone.0153627.

Li X., Arslan F., Ren Y., Adav SS., Poh KK., Sorokin V., Lee CN., Kleijn D de., Lim SK., Sze SK. 2012. Metabolic adaptation to a disruption in oxygen supply during myocardial ischemia and reperfusion is underpinned by temporal and quantitative changes in the cardiac proteome. *Journal of Proteome Research* 11:2331–2346. DOI: 10.1021/pr201025m.

McMahon KM., Volpato M., Chi HY., Musiwaro P., Poterlowicz K., Peng Y., Scally AJ., Patterson LH., Phillips RM., Sutton CW. 2012. Characterization of changes in the proteome in different regions of 3D multicell tumor spheroids. *Journal of Proteome Research* 11:2863–2875. DOI: 10.1021/pr2012472.

Morrison BJ., Hastie ML., Grewal YS., Bruce ZC., Schmidt C., Reynolds BA., Gorman JJ., Lopez JA. 2012. Proteomic comparison of MCF-7 tumoursphere and monolayer cultures. *PLoS ONE* 7:e52692. DOI: 10.1371/journal.pone.0052692.

Rajcevic U., Knol J., Piersma S., Bougnaud S., Fack F., Sundlisaeter E., Sondenaa K., Myklebust R., Pham T., Niclou S., Jimenez C. 2014. Colorectal cancer derived organotypic spheroids maintain essential tissue characteristics but adapt their metabolism in culture. *Proteome Science* 12:39. DOI: 10.1186/1477-5956-12-39.

Ren Y., Hao P., Dutta B., Cheow ESH., Sim KH., Gan CS., Lim SK., Sze SK. 2013. Hypoxia modulates A431 cellular pathways association to tumor radioresistance and enhanced migration revealed by comprehensive proteomic and functional studies. *Molecular & Cellular Proteomics* 12:485–498. DOI: 10.1074/mcp.M112.018325.

Riis S., Stensballe A., Emmersen J., Pennisi CP., Birkelund S., Zachar V., Fink T. 2016. Mass spectrometry analysis of adipose-derived stem cells reveals a significant effect of hypoxia on pathways regulating extracellular matrix. *Stem Cell Research & Therapy* 7:1–14. DOI: 10.1186/s13287-016-0310-7.

Villeneuve L., Tiede LM., Morsey B., Fox HS. 2013. Quantitative proteomics reveals oxygen-dependent changes in neuronal mitochondria affecting function and sensitivity to rotenone. *Journal of Proteome Research* 12:4599–4606. DOI: 10.1021/pr400758d.

Wrzesinski K., Rogowska-Wrzesinska A., Kanlaya R., Borkowski K., Schwämmle V., Dai J., Joensen KE., Wojdyla K., Carvalho VB., Fey SJ. 2014. The cultural divide: Exponential growth in classical 2D and metabolic equilibrium in 3D environments. *PLoS ONE* 9:1–15. DOI: 10.1371/journal.pone.0106973.

Xu Z-W., Chen X., Jin X-H., Meng X-Y., Zhou X., Fan F-X., Mao S-Y., Wang Y., Zhang W-C., Shan N-N., Li Y-M., Xu R-C. 2016. SILAC-based proteomic analysis reveals that salidroside antagonizes cobalt chloride-induced hypoxic effects by restoring the tricarboxylic acid cycle in cardiomyocytes. *Journal of Proteomics* 130:211–220. DOI: 10.1016/j.jprot.2015.09.028.

Yue X., Lukowski JK., Weaver EM., Skube SB., Hummon AB. 2016. Quantitative proteomic and phosphoproteomic comparison of 2D and 3D colon cancer cell culture models. *Journal of Proteome Research* 15:4265–4276. DOI: 10.1021/acs.jproteome.6b00342.
