# Supplementary material for: Chemical composition and the potential for proteomic transformation in cancer, hypoxia, and hyperosmotic stress
Source: PeerJ. 2017 Jun 6;5:e3421. doi: 10.7717/peerj.3421 (PMC5463988; doi:10.7717/peerj.3421)
Supplement: Dataset S1 [file peerj-05-3421-s001.zip › canprot_0.0.5.tar.gz!/canprot/inst/doc/osmotic.html]

Hyperosmotic Stress


# Hyperosmotic Stress

This vignette from the R package canprot reproduces calculations of compositional oxidation state and hydration state that are described in a preprint posted on bioRxiv (Dick, 2017).

## Abbreviations

VHG (very high glucose), ARPE-19 (human retinal pigmented epithelium cells), ECO57 (*Escherichia coli* O157:H7 Sakai), IOBA-NHC (human conjunctival epithelial cells), CAUCR (*Caulobacter crescentus*), tr. (transcriptome), pr. (proteome), CHO (Chinese hamster ovary cells).

## Data Sources

`PW08` VHG (300 g/L) vs control (20 g/L). The comparisons here use proteins with expression ratios < 0.9 or > 1.1 and with p-values < 0.05. Source: SI Table of Pham & Wright (2008).

`WCM+09` 24 h at 16.7 mM vs 5.6 mM glucose. Source: extracted from Suppl. Table ST4 of Waanders et al. (2009); including the red- and blue-highlighted rows in the source table (those with ANOVA *p*-value < 0.01), and applying the authors’ criterion that proteins be identified by 2 or more unique peptides in at least 4 of the 8 most intense LC-MS/MS runs.

`OBBH11` 300 mOsm (control) or 400 mOsm (NaCl treatment). Source: Suppl. Table 1 of Oswald et al. (2011).

`CCC+12` Mannitol-balanced 5.5 (control), 25 or 100 mM ᴅ-glucose media. Source: Table 1 of Chen et al. (2012).

`KKG+12` Temperature and NaCl treatment (control: 35 °C, *a*_w_ = 0.993). Source: Suppl. Tables S13–S16 of Kocharunchitt et al. (2012).

`CCCC13` 5.5 (control), 25 or 100 mM ᴅ-glucose. Source: Table 1 of Chen et al. (2013).

`TSZ+13` Gill proteome of Japanese eel (*Anguilla japonica*) adapted to seawater or freshwater. Source: Protein IDs from Suppl. Table 3 and gene names of human orthologs from Suppl. File 4 of Tse et al. (2013).

`GSC14` 30 min in YNB (2% glucose) vs YPKG (0.5% glucose) media. Source: extracted from Suppl. Files 3 and 5 of Giardina, Stanley & Chiang (2014), using the authors’ criterion of *p*-value <0.05.

`CLG+15` 280 (control), 380, or 480 mOsm (NaCl treatment) for 24 h. Source: Table 2 of Chen et al. (2015).

`KLB+15` Overnight treatment with a final concentration of 40/50 mM NaCl or 200 mM sucrose vs M2 minimal salts medium plus glucose (control). Source: Additional file Table S2 of Kohler et al. (2015).

`LDB+15` 15 g/L vs 5 g/L (control) glucose at days 0, 3, 6, and 9. The comparisons here use all proteins reported to have expression patterns in Cluster 1 (up) or Cluster 5 (down), or only the proteins with high expression differences (ratio ≤-0.2 or ≥0.2) at all time points. Source: SI Table S4 of Liu et al. (2015).

`YDZ+15` 4.21 osmol/kg vs 3.17 osmol/kg osmotic pressure (NaCl treatment). Source: Table 1 of Yang et al. (2015).

`RBP+16` 0.1 M KCl (treatment) vs medium with no added KCl (control). Source: Suppl. Tables 2 and 3 of Silva Rodrigues et al. (2016).

## Summary Table

This table compares the chemical compositions of groups of proteins that are relatively down- and up-expressed (`n1` and `n2`, respectively) in cells grown in hyperosmotic stress compared to control conditions.

```
library(canprot)
data(canprot)
```

```
datasets <- pdat_osmotic()
```

```
comptab <- lapply_canprot(datasets, function(dataset) {
  pdat <- get_pdat(dataset, "pdat_osmotic")
  ZC_nH2O(pdat, plot.it=FALSE)
}, varlist="pdat_osmotic")
```

```
library(xtable)
xsummary(comptab)
```


|  | | | | *Z*_C_** | | | *n*_H2O_** | | |
| --- | --- | --- | --- | --- | --- | --- | --- | --- | --- |
| set | reference (description) | *n*_1_ | *n*_2_ | MD | ES | *p*-value | MD | ES | *p*-value |
| a | `PW08` (S. cerevisiae VHG 2h) | 38 | 44 | -0.010 | 44 | 3e-01 | -0.029 | **39** | 1e-01 |
| b | `PW08` (S. cerevisiae VHG 10h) | 33 | 62 | 0.001 | 51 | 9e-01 | -0.020 | **40** | 1e-01 |
| c | `PW08` (S. cerevisiae VHG 12h) | 18 | 65 | 0.008 | 57 | 4e-01 | -0.017 | 42 | 3e-01 |
| d | `WCM+09` (mouse pancreatic islets) | 63 | 94 | -0.007 | 46 | 4e-01 | **-0.038** | **40** | **4e-02** |
| e | `OBBH11` (adipose-derived stem cells) | 148 | 144 | -0.006 | 46 | 2e-01 | 0.021 | 57 | **5e-02** |
| f | `CCC+12` (ARPE-19 25mM) | 17 | 11 | -0.046 | **32** | 1e-01 | -0.052 | **34** | 2e-01 |
| g | `CCC+12` (ARPE-19 100mM) | 21 | 24 | -0.011 | 48 | 8e-01 | -0.023 | 43 | 4e-01 |
| h | `KKG+12` (ECO57 25C\_aw0.985) | 114 | 61 | 0.002 | 53 | 5e-01 | -0.016 | 45 | 3e-01 |
| i | `KKG+12` (ECO57 14C\_aw0.985) | 238 | 61 | -0.008 | 49 | 8e-01 | -0.025 | 42 | **5e-02** |
| j | `KKG+12` (ECO57 25C\_aw0.967) | 263 | 56 | -0.003 | 47 | 5e-01 | 0.032 | 59 | **3e-02** |
| k | `KKG+12` (ECO57 14C\_aw0.967) | 372 | 73 | -0.002 | 49 | 7e-01 | -0.008 | 48 | 6e-01 |
| l | `CCCC13` (Chang liver cells 25mM) | 32 | 39 | -0.014 | 45 | 4e-01 | 0.001 | 49 | 9e-01 |
| m | `CCCC13` (Chang liver cells 100mM) | 19 | 50 | 0.022 | **61** | 2e-01 | -0.029 | **40** | 2e-01 |
| n | `TSZ+13` (eel gill) | 49 | 28 | 0.000 | 55 | 4e-01 | -0.026 | 43 | 3e-01 |
| o | `GSC14` (S. cerevisiae t30a) | 78 | 77 | 0.003 | 53 | 5e-01 | -0.024 | 41 | 7e-02 |
| p | `GSC14` (S. cerevisiae t30b) | 67 | 67 | -0.002 | 49 | 9e-01 | -0.017 | 45 | 3e-01 |
| q | `GSC14` (S. cerevisiae t30c) | 87 | 87 | -0.001 | 47 | 6e-01 | -0.014 | 45 | 2e-01 |
| r | `CLG+15` (IOBA-NHC) | 25 | 38 | -0.012 | **40** | 2e-01 | 0.010 | 53 | 7e-01 |
| s | `KLB+15` (CAUCR succinate tr.) | 105 | 96 | **0.022** | **63** | **1e-03** | **-0.060** | **35** | **3e-04** |
| t | `KLB+15` (CAUCR NaCl tr.) | 209 | 142 | 0.007 | 56 | **5e-02** | -0.040 | 41 | **3e-03** |
| u | `KLB+15` (CAUCR succinate pr.) | 33 | 33 | **0.019** | **65** | **3e-02** | **-0.045** | **35** | **4e-02** |
| v | `KLB+15` (CAUCR NaCl pr.) | 33 | 27 | 0.018 | **65** | 5e-02 | -0.040 | **36** | 7e-02 |
| w | `LDB+15` (CHO all) | 294 | 205 | **-0.027** | **36** | **3e-07** | -0.020 | 46 | 1e-01 |
| x | `LDB+15` (CHO high) | 66 | 75 | **-0.032** | **35** | **3e-03** | -0.003 | 52 | 7e-01 |
| y | `YDZ+15` (Yarrowia lipolytica) | 14 | 28 | 0.033 | **61** | 2e-01 | -0.032 | 42 | 4e-01 |
| z | `RBP+16` (Paracoccidioides lutzii) | 160 | 141 | 0.002 | 52 | 5e-01 | **-0.044** | **36** | **1e-05** |

## Mean Differences

The dataset for adipose-derived stem cells is highlighted in orange.

```
col <- rep("black", length(datasets))
col[grepl("=ASC", datasets)] <- "orange"
diffplot(comptab, col=col)
```

## References

Chen Y-H., Chen J-Y., Chen Y-W., Lin S-T., Chan H-L. 2012. High glucose-induced proteome alterations in retinal pigmented epithelium cells and its possible relevance to diabetic retinopathy. *Molecular Biosystems* 8:3107–3124. DOI: 10.1039/C2MB25331C.

Chen J-Y., Chou H-C., Chen Y-H., Chan H-L. 2013. High glucose-induced proteome alterations in hepatocytes and its possible relevance to diabetic liver disease. *Journal of Nutritional Biochemistry* 24:1889–1910. DOI: 10.1016/j.jnutbio.2013.05.006.

Chen L., Li J., Guo T., Ghosh S., Koh SK., Tian D., Zhang L., Jia D., Beuerman RW., Aebersold R., Chan ECY., Zhou L. 2015. Global metabonomic and proteomic analysis of human conjunctival epithelial cells (IOBA-NHC) in response to hyperosmotic stress. *Journal of Proteome Research* 14:3982–3995. DOI: 10.1021/acs.jproteome.5b00443.

Giardina BJ., Stanley BA., Chiang H-L. 2014. Glucose induces rapid changes in the secretome of *Saccharomyces cerevisiae*. *Proteome Science* 12:9. DOI: 10.1186/1477-5956-12-9.

Kocharunchitt C., King T., Gobius K., Bowman JP., Ross T. 2012. Integrated transcriptomic and proteomic analysis of the physiological response of *Escherichia coli* O157:H7 Sakai to steady-state conditions of cold and water activity stress. *Molecular & Cellular Proteomics* 11:M111.009019. DOI: 10.1074/mcp.M111.009019.

Kohler C., Lourenço RF., Bernhardt J., Albrecht D., Schüler J., Hecker M., Gomes SL. 2015. A comprehensive genomic, transcriptomic and proteomic analysis of a hyperosmotic stress sensitive \(\alpha\)-proteobacterium. *BMC Microbiology* 15:1–15. DOI: 10.1186/s12866-015-0404-x.

Liu Z., Dai S., Bones J., Ray S., Cha S., Karger BL., Li JJ., Wilson L., Hinckle G., Rossomando A. 2015. A quantitative proteomic analysis of cellular responses to high glucose media in Chinese hamster ovary cells. *Biotechnology Progress* 31:1026–1038. DOI: 10.1002/btpr.2090.

Oswald ES., Brown LM., Bulinski JC., Hung CT. 2011. Label-free protein profiling of adipose-derived human stem cells under hyperosmotic treatment. *Journal of Proteome Research* 10:3050–3059. DOI: 10.1021/pr200030v.

Pham TK., Wright PC. 2008. The proteomic response of *Saccharomyces cerevisiae* in very high glucose conditions with amino acid supplementation. *Journal of Proteome Research* 7:4766–4774. DOI: 10.1021/pr800331s.

Silva Rodrigues LN da., Almeida Brito W de., Parente AFA., Weber SS., Bailão AM., Casaletti L., Borges CL., Almeida Soares CM de. 2016. Osmotic stress adaptation of *Paracoccidioides lutzii*, Pb01, monitored by proteomics. *Fungal Genetics and Biology* 95:13–23. DOI: 10.1016/j.fgb.2016.08.001.

Tse WKF., Sun J., Zhang H., Law AYS., Yeung BHY., Chow SC., Qiu J-W., Wong CKC. 2013. Transcriptomic and iTRAQ proteomic approaches reveal novel short-term hyperosmotic stress responsive proteins in the gill of the Japanese eel (*Anguilla japonica*). *Journal of Proteomics* 89:81–94. DOI: 10.1016/j.jprot.2013.05.026.

Waanders LF., Chwalek K., Monetti M., Kumar C., Lammert E., Mann M. 2009. Quantitative proteomic analysis of single pancreatic islets. *Proceedings of the National Academy of Sciences* 106:18902–18907. DOI: 10.1073/pnas.0908351106.

Yang L-B., Dai X-M., Zheng Z-Y., Zhu L., Zhan X-B., Lin C-C. 2015. Proteomic analysis of erythritol-producing *Yarrowia lipolytica* from glycerol in response to osmotic pressure. *Journal of Microbiology and Biotechnology* 25:1056–1069. DOI: 10.4014/jmb.1412.12026.
