# Supplementary material for: Chemical composition and the potential for proteomic transformation in cancer, hypoxia, and hyperosmotic stress
Source: PeerJ. 2017 Jun 6;5:e3421. doi: 10.7717/peerj.3421 (PMC5463988; doi:10.7717/peerj.3421)
Supplement: Dataset S1 [file peerj-05-3421-s001.zip › canprot_0.0.5.tar.gz!/canprot/inst/doc/pancreatic.html]

Pancreatic Cancer


# Pancreatic Cancer

This vignette from the R package canprot reproduces calculations of compositional oxidation state and hydration state that are described in a preprint posted on bioRxiv (Dick, 2017).

## Abbreviations

T (tumor), N (normal), CP (chronic pancreatitis), AIP (autoimmune pancreatitis), PC (pancreatic cancer), DM (diabetes mellitus), PDAC (pancreatic ductal adenocarcinoma), ANT (adjacent normal tissue), FFPE (Formalin-fixed paraffin-embedded), LCM (laser-capture microdissection), NP (normal pancreas).

## Data Sources

`LHE+04` Pooled tissue samples of PC and matched normal tissue from 12 patients. Source: Tables 2 and 3 of Lu et al. (2004).

`CYD+05` Two PC and two normal pancreas samples. Source: Tables 1 and 2 of Chen et al. (2005).

`CGB+05` Large-scale immunoblotting (PowerBlot) of 8 tissue specimens of pancreatic intraepithelial neoplasia compared to normal pancreas and CP. Source: Table 2 of Crnogorac-Jurcevic et al. (2005).

`CBP+07` Tissue specimens from patients with CP (without any findings of pancreatic cancer) and 10 control specimens from patients with normal pancreas. Source: Table 1 of Chen et al. (2007).

`CTZ+09` 12 carcinoma samples (PDAC), 12 benign pancreatic adenocarcinomas and 10 normal tissues adjacent to the PDAC primary mass. Source: Table 1 of Cui et al. (2009).

`MLC+11` Source: extracted from Suppl. Table S2 of McKinney et al. (2011).

`PCS+11` PDAC compared to normal pancreas. Source: Suppl. Table 3 of Pan et al. (2011).

`TMW+11` Potentially accessible proteins in fresh samples of PC tumors (three patients) vs normal tissue (two patients with normal pancreas and one with CP). Source: extracted from the SI Table of Turtoi et al. (2011).

`KBK+12` 11 tissue specimens containing >50% cancer and 8 unmatched, uninvolved tissues adjacent to pancreatitis. Source: Suppl. Tables 2 and 3 of Kojima et al. (2012).

`KHO+13` Fresh-frozen PDAC tissue specimens from 7 patients vs a pooled mixture of 3 normal main pancreatic duct tissue samples. Source: extracted from SI Table S3 of Kawahara et al. (2013), including proteins with an expression ratio >2 [or <0.5] in at least 5 of the 7 experiments and ratio >1 [or <1] in all experiments.

`KPC+13` Frozen samples of PDAC tumors vs adjacent benign tissue from four patients. Source: Suppl. Table 2 of Kosanam et al. (2013).

`PKB+13` Tissue samples from 3 patients with PC vs 3 patients with AIP or 3 patients with CP. Source: extracted from Tables 2, 3, and 4 of Paulo et al. (2013).

`WLL+13` 12 samples each (pooled) of low-grade tumor or high-grade tumor vs non-tumor. Source: extracted from Suppl. Tables S4 and S5 of Wang et al. (2013a), including proteins with ratios ≥1.5 or ≤0.667 for at least 2 of the 4 groups, and with expression differences for all 4 groups in the same direction.

`WLL+13a` Source: extracted from Suppl. Tables S3 and S4 of Wang et al. (2013b), including proteins with >3/2 or <2/3 fold change in at least 3 of 4 iTRAQ experiments for different pooled samples.

`ZNWL13` LCM of CD24^+^ cells from PDAC vs CD24^-^ cells from adjacent normal tissue (ANT). Source: SI Table S5 of Zhu et al. (2013).

`ISI+14` Matched PDAC and normal tissue from nine patients. Source: extracted from SI Table S5 of Iuga et al. (2014), excluding proteins marked as “not passed”, i.e. having inconsistent regulation.

`KKC+16` PDAC tumors in transgenic mice vs pancreas in normal mice, analyzed at time points of 2.5, 3.5, 5 and 10 weeks. Source: Suppl. Table of Kuo et al. (2016).

## Summary Table

This table compares the chemical compositions of groups of human proteins that are relatively down- and up-expressed (`n1` and `n2`, respectively) in pancreatic cancer compared to non-tumor tissue.

```
library(canprot)
data(canprot)
```

```
datasets <- pdat_pancreatic()
```

```
comptab <- lapply_canprot(datasets, function(dataset) {
  pdat <- get_pdat(dataset, "pdat_pancreatic")
  ZC_nH2O(pdat, plot.it=FALSE)
}, varlist="pdat_pancreatic")
```

```
library(xtable)
xsummary(comptab)
```


|  | | | | *Z*_C_** | | | *n*_H2O_** | | |
| --- | --- | --- | --- | --- | --- | --- | --- | --- | --- |
| set | reference (description) | *n*_1_ | *n*_2_ | MD | ES | *p*-value | MD | ES | *p*-value |
| a | `LHE+04` (T / N) | 41 | 69 | 0.002 | 53 | 6e-01 | **0.062** | **66** | **5e-03** |
| b | `CYD+05` (T / N) | 60 | 88 | **0.025** | **61** | **2e-02** | -0.009 | 48 | 7e-01 |
| c | `CGB+05` (T / N) | 48 | 54 | 0.015 | 59 | 1e-01 | -0.014 | 48 | 8e-01 |
| d | `CBP+07` (CP / N) | 19 | 95 | 0.009 | 54 | 6e-01 | **-0.053** | **30** | **6e-03** |
| e | `CTZ+09` (T / N) | 28 | 29 | 0.023 | **64** | 8e-02 | 0.027 | 58 | 3e-01 |
| f | `MLC+11` (T / N) | 38 | 45 | 0.021 | 56 | 4e-01 | 0.015 | 58 | 2e-01 |
| g | `PCS+11` (FFPE T / N) | 207 | 152 | **0.042** | **69** | **2e-09** | -0.018 | 45 | 1e-01 |
| h | `TMW+11` (accessible T / N) | 108 | 86 | **-0.031** | **35** | **5e-04** | -0.026 | 41 | **4e-02** |
| i | `KBK+12` (FFPE T / N) | 38 | 47 | **0.039** | **69** | **3e-03** | 0.024 | 59 | 1e-01 |
| j | `KHO+13` (T / N) | 78 | 57 | -0.011 | 43 | 2e-01 | **0.041** | **60** | **5e-02** |
| k | `KPC+13` (T / N) | 257 | 456 | 0.006 | 52 | 5e-01 | **0.036** | **60** | **2e-05** |
| l | `PKB+13` (FFPE PC / AIP) | 29 | 73 | -0.015 | 42 | 2e-01 | 0.023 | 56 | 3e-01 |
| m | `PKB+13` (FFPE PC / CP) | 53 | 73 | -0.016 | 45 | 3e-01 | **0.054** | **66** | **2e-03** |
| n | `WLL+13` (low-grade T / N) | 83 | 32 | 0.008 | 58 | 2e-01 | -0.036 | **40** | 1e-01 |
| o | `WLL+13` (high-grade T / N) | 224 | 176 | **0.022** | **62** | **3e-05** | -0.003 | 49 | 7e-01 |
| p | `WLL+13a` (T / N (no DM)) | 208 | 219 | **0.034** | **68** | **1e-10** | 0.000 | 50 | 9e-01 |
| q | `WLL+13a` (T / N (DM)) | 56 | 167 | **0.034** | **66** | **4e-04** | **0.040** | **63** | **4e-03** |
| r | `ZNWL13` (LCM PDAC / ANT) | 227 | 148 | **0.035** | **67** | **1e-08** | -0.007 | 49 | 6e-01 |
| s | `ISI+14` (T / N) | 65 | 34 | 0.002 | 53 | 6e-01 | **-0.050** | **36** | **3e-02** |
| t | `KKC+16` (mouse 2.5 w T / N) | 35 | 51 | -0.015 | 44 | 4e-01 | -0.004 | 46 | 6e-01 |
| u | `KKC+16` (mouse 3.5 w T / N) | 40 | 73 | -0.019 | 42 | 1e-01 | **0.045** | **65** | **1e-02** |
| v | `KKC+16` (mouse 5 w T / N) | 49 | 84 | 0.015 | 57 | 2e-01 | -0.005 | 49 | 9e-01 |
| w | `KKC+16` (mouse 10 w T / N) | 37 | 108 | 0.014 | 58 | 2e-01 | 0.024 | 58 | 2e-01 |

## Mean Differences

```
col <- rep("black", length(datasets))
col[grepl("=mouse", datasets)] <- "red"
diffplot(comptab, col=col)
```

## References

Chen R., Yi EC., Donohoe S., Pan S., Eng J., Cooke K., Crispin DA., Lane Z., Goodlett DR., Bronner MP., Aebersold R., Brentnall TA. 2005. Pancreatic cancer proteome: The proteins that underlie invasion, metastasis, and immunologic escape. *Gastroenterology* 129:1187–1197. DOI: 10.1053/j.gastro.2005.08.001.

Chen R., Brentnall TA., Pan S., Cooke K., Moyes KW., Lane Z., Crispin DA., Goodlett DR., Aebersold R., Bronner MP. 2007. Quantitative proteomics analysis reveals that proteins differentially expressed in chronic pancreatitis are also frequently involved in pancreatic cancer. *Molecular & Cellular Proteomics* 6:1331–1342. DOI: 10.1074/mcp.M700072-MCP200.

Crnogorac-Jurcevic T., Gangeswaran R., Bhakta V., Capurso G., Lattimore S., Akada M., Sunamura M., Prime W., Campbell F., Brentnall TA., Costello E., Neoptolemos J., Lemoine NR. 2005. Proteomic analysis of chronic pancreatitis and pancreatic adenocarcinoma. *Gastroenterology* 129:1454–1463. DOI: 10.1053/j.gastro.2005.08.012.

Cui Y., Tian M., Zong M., Teng M., Chen Y., Lu J., Jiang J., Liu X., Han J. 2009. Proteomic analysis of pancreatic ductal adenocarcinoma compared with normal adjacent pancreatic tissue and pancreatic benign cystadenoma. *Pancreatology* 9:89–98. DOI: 10.1159/000178879.

Iuga C., Seicean A., Iancu C., Buiga R., Sappa PK., Völker U., Hammer E. 2014. Proteomic identification of potential prognostic biomarkers in resectable pancreatic ductal adenocarcinoma. *Proteomics* 14:945–955. DOI: 10.1002/pmic.201300402.

Kawahara T., Hotta N., Ozawa Y., Kato S., Kano K., Yokoyama Y., Nagino M., Takahashi T., Yanagisawa K. 2013. Quantitative proteomic profiling identifies DPYSL3 as pancreatic ductal adenocarcinoma-associated molecule that regulates cell adhesion and migration by stabilization of focal adhesion complex. *PLoS ONE* 8:e79654. DOI: 10.1371/journal.pone.0079654.

Kojima K., Bowersock GJ., Kojima C., Klug CA., Grizzle WE., Mobley JA. 2012. Validation of a robust proteomic analysis carried out on formalin-fixed paraffin-embedded tissues of the pancreas obtained from mouse and human. *Proteomics* 12:3393–3402. DOI: 10.1002/pmic.201100663.

Kosanam H., Prassas I., Chrystoja CC., Soleas I., Chan A., Dimitromanolakis A., Blasutig IM., Rückert F., Gruetzmann R., Pilarsky C., Maekawa M., Brand R., Diamandis EP. 2013. Laminin, gamma 2 (LAMC2): A promising new putative pancreatic cancer biomarker identified by proteomic analysis of pancreatic adenocarcinoma tissues. *Molecular & Cellular Proteomics* 12:2820–2832. DOI: 10.1074/mcp.M112.023507.

Kuo K-K., Kuo C-J., Chiu C-Y., Liang S-S., Huang C-H., Chi S-W., Tsai K-B., Chen C-Y., Hsi E., Cheng K-H., Chiou S-H. 2016. Quantitative proteomic analysis of differentially expressed protein profiles involved in pancreatic ductal adenocarcinoma. *Pancreas* 45:71–83. DOI: 10.1097/MPA.0000000000000388.

Lu Z., Hu L., Evers S., Chen J., Shen Y. 2004. Differential expression profiling of human pancreatic adenocarcinoma and healthy pancreatic tissue. *Proteomics* 4:3975–3988. DOI: 10.1002/pmic.200300863.

McKinney KQ., Lee Y-Y., Choi H-S., Groseclose G., Iannitti DA., Martinie JB., Russo MW., Lundgren DH., Han DK., Bonkovsky HL., Hwang S-I. 2011. Discovery of putative pancreatic cancer biomarkers using subcellular proteomics. *Journal of Proteomics* 74:79–88. DOI: 10.1016/j.jprot.2010.08.006.

Pan S., Chen R., Stevens T., Bronner MP., May D., Tamura Y., McIntosh MW., Brentnall TA. 2011. Proteomics portrait of archival lesions of chronic pancreatitis. *PLoS ONE* 6:1–12. DOI: 10.1371/journal.pone.0027574.

Paulo JA., Kadiyala V., Brizard S., Banks PA., Steen H., Conwell DL. 2013. A proteomic comparison of formalin-fixed paraffin-embedded pancreatic tissue from autoimmune pancreatitis, chronic pancreatitis, and pancreatic cancer. *Journal of the Pancreas* 14:405–414. DOI: 10.6092/1590-8577/1508.

Turtoi A., Musmeci D., Wang Y., Dumont B., Somja J., Bevilacqua G., De Pauw E., Delvenne P., Castronovo V. 2011. Identification of novel accessible proteins bearing diagnostic and therapeutic potential in human pancreatic ductal adenocarcinoma. *Journal of Proteome Research* 10:4302–4313. DOI: 10.1021/pr200527z.

Wang W-S., Liu X-H., Liu L-X., Lou W-H., Jin D-Y., Yang P-Y., Wang X-L. 2013a. ITRAQ-based quantitative proteomics reveals myoferlin as a novel prognostic predictor in pancreatic adenocarcinoma. *Journal of Proteomics* 91:453–465. DOI: 10.1016/j.jprot.2013.06.032.

Wang W-S., Liu X-H., Liu L-X., Jin D-Y., Yang P-Y., Wang X-L. 2013b. Identification of proteins implicated in the development of pancreatic cancer-associated diabetes mellitus by iTRAQ-based quantitative proteomics. *Journal of Proteomics* 84:52–60. DOI: 10.1016/j.jprot.2013.03.031.

Zhu J., Nie S., Wu J., Lubman DM. 2013. Target proteomic profiling of frozen pancreatic CD24+ adenocarcinoma tissues by immuno-laser capture microdissection and nano-LC–MS/MS. *Journal of Proteome Research* 12:2791–2804. DOI: 10.1021/pr400139c.
